# Comparing Ultraconserved Elements and Exons for Phylogenomic Analyses of Middle American Cichlids: When Data Agree to Disagree

**DOI:** 10.1093/gbe/evab161

**Published:** 2021-07-17

**Authors:** Fernando Alda, William B Ludt, Diego J Elías, Caleb D McMahan, Prosanta Chakrabarty

**Affiliations:** 1 Department of Biology, Geology and Environmental Science, University of Tennessee at Chattanooga, Tennessee, USA; 2 Department of Ichthyology, Natural History Museum of Los Angeles County, Los Angeles, California, USA; 3 Museum of Natural Science, Department of Biological Sciences, Louisiana State University, Baton Rouge, Louisiana, USA; 4 Field Museum of Natural History, Chicago, Illinois, USA

**Keywords:** gene tree heterogeneity, Heroini, hybrid target capture, phylogenetic informativeness, signal–noise ratio, species trees

## Abstract

Choosing among types of genomic markers to be used in a phylogenomic study can have a major influence on the cost, design, and results of a study. Yet few attempts have been made to compare categories of next-generation sequence markers limiting our ability to compare the suitability of these different genomic fragment types. Here, we explore properties of different genomic markers to find if they vary in the accuracy of component phylogenetic trees and to clarify the causes of conflict obtained from different data sets or inference methods. As a test case, we explore the causes of discordance between phylogenetic hypotheses obtained using a novel data set of ultraconserved elements (UCEs) and a recently published exon data set of the cichlid tribe Heroini. Resolving relationships among heroine cichlids has historically been difficult, and the processes of colonization and diversification in Middle America and the Greater Antilles are not yet well understood. Despite differences in informativeness and levels of gene tree discordance between UCEs and exons, the resulting phylogenomic hypotheses generally agree on most relationships. The independent data sets disagreed in areas with low phylogenetic signal that were overwhelmed by incomplete lineage sorting and nonphylogenetic signals. For UCEs, high levels of incomplete lineage sorting were found to be the major cause of gene tree discordance, whereas, for exons, nonphylogenetic signal is most likely caused by a reduced number of highly informative loci. This paucity of informative loci in exons might be due to heterogeneous substitution rates that are problematic to model (i.e., computationally restrictive) resulting in systematic errors that UCEs (being less informative individually but more uniform) are less prone to. These results generally demonstrate the robustness of phylogenomic methods to accommodate genomic markers with different biological and phylogenetic properties. However, we identify common and unique pitfalls of different categories of genomic fragments when inferring enigmatic phylogenetic relationships.

Significance

Phylogenomic data sets often consist of reduced representations of the genome, but differences inherent to the nature of these genomic markers may affect their performance. The information content of individual genomic markers is rarely interrogated, let alone compared with different types of markers from target-capture sequencing methods. This information will be important for deciding which data types are better suited for resolving the relationships of interest. We explored the causes of phylogenetic disagreement in the relationships of heroine cichlids by comparing topologies, support, phylogenetic informativeness, gene tree heterogeneity, and other sources of discordance based on ultraconserved elements and exons. We found that both types of genomic markers are affected by low phylogenetic signal-to-noise ratios, which for ultraconserved elements might be amplified by extensive incomplete lineage sorting and for exons by systematic errors from overly generalized model-fitting. More accurate modeling of substitution rates from individual loci, and of dealing with incomplete lineage sorting, will likely increase the accuracy of phylogenomic trees and resolve the discordance between data types in the future.

## Introduction

Collecting massive amounts of genomic data is no longer a limiting factor for molecular evolutionary studies of nonmodel organisms, thanks largely to the great advances and cost reduction of massive parallel sequencing technologies (Goodwin et al. 2016). The increased power for the analysis of hundreds, to thousands, of independent genomic markers has shed light on complex evolutionary histories and provided reliable well-supported trees (Crawford et al. 2012; Jarvis et al. 2014; Wickett et al. 2014; Irisarri et al. 2017; Hughes et al. 2018; Williams et al. 2020). In contrast, it has been concurrently evidenced that some nodes in the Tree of Life are inevitably difficult to resolve despite increasing amounts of data (Philippe et al. 2011; Arcila et al. 2017; Chakrabarty et al. 2017; Shen et al. 2017; Walker et al. 2018; Alda et al. 2019). These studies have contributed to a deeper understanding of the complications that come along with having an abundance of genomic data, namely: 1) the influence of gene tree heterogeneity including the disproportionate effect of individual loci; and 2) how overly generalized models impact phylogenetic inference of species trees (Smith et al. 2015; Walker et al. 2018; Bravo et al. 2019).

The type of molecular marker used for phylogenomic analyses likely plays a major role in any of the potential pitfalls mentioned above. Therefore, choosing how or what loci to sample across the genome is a critical step toward accurately reconstructing the evolutionary history of species or populations, and should be dependent on the biological question of interest (Salichos and Rokas 2013; Chen et al. 2015; Reddy et al. 2017). Ideally, the selection of markers would be carried out following the generation of complete genome data; this a posteriori approach would allow for the selection of loci with the desired properties for the taxonomic scale of interest (e.g., phylogenetic informativeness [PI]; Townsend 2007). Unfortunately, limited time and funding seldom allow for comparing different data sets before undertaking a phylogenomic study, and the choice of markers is more often carried out a priori and driven by convenience and cost than by their specific features (Bravo et al. 2019). Nevertheless, the exploration of phylogenomic data sets is of great utility for evaluating data quality, biological properties, and phylogenetic signal, which translate in their power for consistently resolving relationships (Salichos and Rokas 2013; Reddy et al. 2017; Alda et al. 2019; Burbrink et al. 2020). Recently, it has become increasingly common to interrogate the data used in phylogenetic reconstruction to explore whether data sets contain sufficient unambiguous information to accurately infer species trees (Doyle et al. 2015; Arcila et al. 2017; Alda et al. 2019; Burbrink et al. 2020). Yet, most of these studies only explore a single data set, subsets of that data set, or only one type of genomic marker (but see Gilbert et al. 2015; Reddy et al. 2017; Betancur-R et al. 2019; Bossert et al. 2019; Karin et al. 2020; Arcila et al. 2021).

Most empirical phylogenomic studies are based on sequences from select subsets of the genome, such as coding sequences—either exons or transcriptomes—or highly conserved regions, like ultraconserved elements (UCEs) (Faircloth et al. 2012; Lemmon and Lemmon 2013; McCormack et al. 2013; Jones and Good 2016). However, among the diversity of genomic marker types, it remains unclear which specific features of each type makes them better suited for the variety of different phylogenetic questions (Chen et al. 2015; Reddy et al. 2017; Karin et al. 2020). Essentially, each marker type brings advantages and disadvantages. For example, the effect of selection on exons can result in base composition heterogeneity and violations of common evolutionary models, including multispecies coalescent methods that assume neutrality (Edwards et al. 2016; Romiguier and Roux 2017). Selection might also lead to decreased effective population size across the genome and, as a consequence, to lower levels of incomplete lineage sorting (ILS) than in noncoding regions (Scally et al. 2012). On the other hand, UCEs are noncoding regions and their functions are not completely understood (Bejerano et al. 2004). There is evidence that UCEs are associated with regulatory functions and are conserved due to purifying selection (Katzman et al. 2007); however, whether they are under strong selective pressure is unclear (Chen et al. 2007). Variation in each UCE locus increases proportionally to the distance from the highly conserved core region. Because of this, UCEs have been proven useful for analyses at both deep and recent evolutionary timescales (Faircloth et al. 2012; Burress et al. 2018). However, the high variation in substitution rates as a function of distance from the conserved core likely results in complex patterns of molecular evolution both within and between loci, increasing the difficulty in obtaining accurate alignments and estimates of their best-fitting evolutionary models (Tagliacollo and Lanfear 2018).

It should not be surprising, given the uncertainty of the variation related to genomic markers, that data sets based on different marker types produce discordant species trees. When this is the case, it can be assumed that one or more types of genomic markers may be less appropriate than others when applied to a particular question or tree (Reddy et al. 2017; Bravo et al. 2019). Despite its limits, phylogenomic inference is quite robust and largely consistent across studies. The relatively few cases of disagreement (e.g., Betancur-R et al. 2019) are most often characterized by short internal branches containing reduced phylogenetic signal that can be easily overwhelmed by ILS, random errors or noise (e.g., homoplasy), and systematic errors (e.g., model violations) that may lead to erroneous topologies despite high support (Rodríguez-Ezpeleta et al. 2007; Kapli et al. 2020; Simion et al. 2020). Ultimately, whether data sets agree or disagree is the result of high- or low signal-to-noise ratios, respectively. Phylogenetic discordance is, therefore, prevalent in rapid radiations because the fast sequence of cladogenetic events, often accompanied by large effective population sizes, might be insufficient for genetic changes to accumulate and for alleles to be fully sorted out, resulting in low signal and high noise levels (Alda et al. 2019).

When feasible, marker selection should be considered in the design of every phylogenomic study. Furthermore, exploring the differences in phylogenetic signal across marker types may reveal the unequal genomic distribution of congruence and incongruence and the biological causes for disagreement among phylogenetic hypotheses (Burbrink et al. 2020).

### Study Case: Neotropical Cichlids (Tribe Heroini)

Many long-standing phylogenetic disputes involve resolving rapidly diversifying radiations, of which some of the most compelling examples are cichlids. Cichlid fishes show remarkable cases of adaptive radiations, parallel evolution, and hybrid speciation, among other classic textbook examples of speciation mechanisms (Brawand et al. 2014; Matschiner et al. 2020). Most studies have focused on the East African species but other clades, such as the Neotropical Cichlinae, have not received as much attention despite their high species richness (López-Fernández et al. 2013; McMahan et al. 2013). Within this subfamily, the tribe Heroini, which holds approximately 150 of the 525 Neotropical cichlid species, is the dominant clade in Middle America (Central America, Mexico, Greater Antilles) and represents a major component of the ichthyological diversity of the region (Smith and Bermingham 2005; Matamoros et al. 2015). Heroine cichlids have also radiated into a much wider spectrum of morphological and ecological diversity than other South American cichlids, likely as a result of new ecological opportunities available following colonization (Arbour and López-Fernández 2016). Yet, the events by which cichlid fishes occupied continental Middle America and the Greater Antilles are not completely understood and multiple hypotheses have been proposed, ranging from dispersal through the Proto-Antillean Arch, the Greater Antillean Aves Ridge land bridge (GAARlandia), the East Margin Corridor, or through the Isthmus of Panama (Chakrabarty 2006; Chakrabarty and Albert 2011; Říčan et al. 2013; Tagliacollo et al. 2017). These hypotheses are based on different area cladograms, and therefore accurately resolving the sequence of cladogenetic events in this radiation is essential to reconstructing the biogeographic history of this group, and ultimately understand major patterns of Neotropical fish diversity.

Several studies have attempted to resolve the phylogenetic relationships of heroine cichlids using a variety of morphological and molecular characters. On one hand, the mixture of extensive morphological diversity and ecologically driven convergence of traits has historically hindered the systematics and taxonomy of heroine cichlids, particularly at the genus level (Chakrabarty 2007; Říčan et al. 2016). On the other hand, molecular studies have evidenced the weakness of the morphological phylogenetic signal by showing strong disagreement with morphological hypotheses, but also among different genetic data sets (Roe et al. 1997; Concheiro Pérez et al. 2007; Říčan et al. 2008, 2013, 2016; McMahan et al. 2015; Ilves et al. 2018).

Despite the majority of molecular studies agreeing on the reciprocal monophyly of the major groups in the tribe Heroini (e.g., herichthyines, caquetaines, amphilophines), there is extensive discordance among the relationships within and between these groups (Hulsey et al. 2010; Říčan et al. 2013, 2016; Tagliacollo et al. 2017; Ilves et al. 2018). Significantly, the alternative relationships recovered for the major clades of Middle and South American Heroini have a profound impact on their biogeographic reconstruction (e.g., the number of colonization events, direction of colonization; Říčan et al. 2013, 2016; Tagliacollo et al. 2017).

Differences among multilocus data sets may be attributed to the stochasticity of sampling a small number of loci with different phylogenetic signals, including cytonuclear discordances, but for genomic-scale data sets, random errors are expected to become less important (Irisarri et al. 2018), and discordant phylogenies, when they occur, are more likely to be caused by systematic errors (Kapli et al. 2020). Differences between the most comprehensive studies in taxonomic and genomic coverage have therefore been attributed to inference methods used (i.e., concatenated vs. coalescence-based), although these have not been formally tested (López-Fernández et al. 2013; Říčan et al. 2016; Ilves et al. 2018).

In this study, we hypothesize that in addition to inference methods, inconsistent evolutionary hypotheses can be caused by differences in the phylogenetic signal of different categories of genomic markers that affects their performance or utility for resolving conflicting relationships. To test our hypothesis, we compare the phylogenetic relationships inferred from analyses of a novel UCE genomic data set with a recently published exon-based phylogenomic study (Ilves et al. 2018). We analyze these different data sets using the same inference methods and explore the causes of disagreement by comparing parameter estimates related to PI and signal-to-noise ratio in each data set. Results from our research clarify the relationships among the major clades of Heroini, further resolving their taxonomy while also uncovering the remaining problems that should be addressed (either with increased genomic or taxonomic sampling, or with novel analytical approaches).

## Results

We sequenced more than 297 million reads with a mean of 3.2 million reads per sample from 83 individuals representing 79 species of cichlids (supplementary table 1, Supplementary Material online). We assembled a mean of 294.6 (SD = 283.9) contigs per sample with an average length of 299.3 (SD = 26.4) bp (supplementary table 1, Supplementary Material online). The resulting 75% complete data matrix included 465 UCE loci, with a mean locus length of 1,024.7 bp (SD = 37.2) and containing 91.5 (SD = 4.8) samples on average per alignment. Following alignment and trimming, the average alignment length was 637.7 bp (SD = 119.7) per locus, resulting in a total length of 296,529 bp for the concatenated alignment. Overall, there were 72,012 polymorphic sites (24.3%), of which 33,652 were parsimony-informative (11.3%) (mean 154.8 polymorphic sites and 72.4 parsimony-informative sites per locus; table 1).

**Table 1:** Summary Measurements of Polymorphism: Mean Length, Parsimony Informative Sites, and Polymorphic Sites Per Locus, Observed for Each Genomic Marker Type (UCEs and Exons) and Set of Heroine Cichlid Species (Complete and Common Taxon Sets)

	No. Loci	Mean Locus Length (SD)	Mean Inf. Sites (SD)	Mean Pol. Sites (SD)	% Inf. Sites	% Pol. Sites
UCEs (complete)	465	637.7 (119.7)	72.4 (40.1)	154.8 (64.9)	11.3	24.3
Total		296,529	33,652	72,012	11.3	24.3
UCEs (common)	465	637.7 (119.6)	53.2 (32.7)	128.8 (61.3)	8.3	20.2
Total		296,529	24,757	59,889		
Exons	415	1,136.0 (345.1)	118.6 (53.3)	269.1 (108.6)	10.5	23.7
Total		471,448	49,233	111,678		

### Phylogenomic Analysis of the Complete Taxon Set of UCE Data

**Fig. 1:**
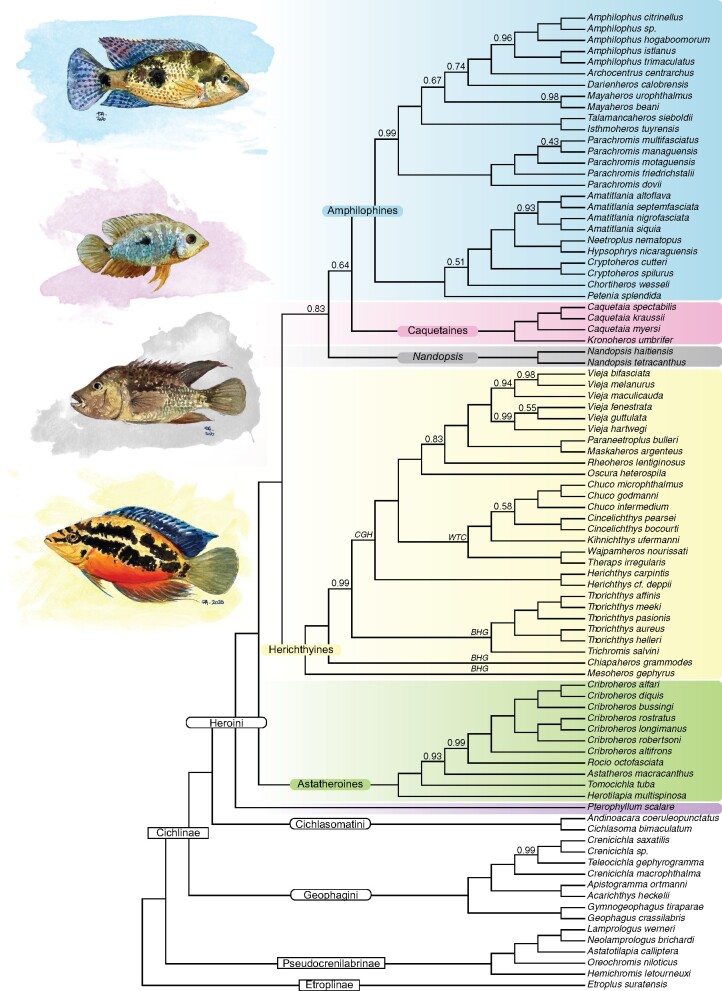
Species tree inferred in ASTRAL-III for the complete data set of UCEs of all cichlid species included in this study. All nodes are supported by local posterior probabilities=1.0 unless otherwise noted. Relevant clades mentioned in the text are indicated: *CGH*, crown-group herichthyines; *BHG*, basal herichthyine genera; *WTC*, *Wajpamheros, Theraps, Chuco* clade. Fish illustrations from top to bottom: *Darienheros calobrensis*, *Caquetaia spectabilis*, *Nandopsis haitiensis*, *Trichromis salvini* (by E. Alda).

The phylogenomic analysis of UCEs from 88 species of cichlids, including 72 species of heroines, resulted in highly supported and largely congruent trees across the different inference methods (fig. 1 and supplementary figs. 1–4, Supplementary Material online). The coalescent-based and concatenated analyses recovered the monophyly of Heroini with high support and each of the major groups within that tribe: amphilophines, herichthyines, astatheroines, caquetaines, and *Nandopsis* (ASTRAL local posterior probabilities = 1.0, maximum likelihood [ML] bootstrap = 100, SVDquartets bootstrap = 100).

Given the overall similarity of the topologies, we considered the ASTRAL-III coalescent species tree (fig. 1) the principal phylogenetic hypothesis. Coalescent-based species trees are expected to yield more accurate tree estimates by directly modeling gene tree heterogeneity and might be preferred over concatenation, particularly when dealing with recently diverged species, with high effective population sizes and high levels of ILS (Irisarri 2020; Kapli et al. 2020). In addition, we considered the ASTRAL tree a more conservative hypothesis because it is less biased toward overestimating bootstrap support values than the concatenation model (Liu et al. 2015). Despite the overall preference for the coalescent approach, we also compare the coalescent-based hypothesis with the other trees obtained, and we will also discuss the potential impact of the type of molecular marker, taxon sampling, and inference method on the recovered topologies.

Within Heroini, we recovered the South American genus *Pterophyllum* as the sister clade to all heroines. The astatheroines were found to be the sister group to all other Middle American heroines (which includes species distributed from southern Mexico to Panama). Astatheroini was recovered as the sister clade to a group comprising herichthyines + amphilophines + caquetaines + *Nandopsis*. In the ASTRAL species tree, we recovered the Middle American amphilophines as the sister group to the South American caquetaines, and together these were the sister clade to the Greater Antillean endemic genus *Nandopsis*. Conversely, in the ML concatenated tree and the SVDquartets coalescent species tree, *Nandopsis* was recovered as the sister clade to herichthyines. Notably, the relationships in either of these topologies only showed moderate to low support (local posterior probability = 0.83–0.64, ML bootstrap = 81–67) (fig. 1 and supplementary fig. 2, Supplementary Material online), except in the case of the SVDquartets analysis were all the nodes showed 100% bootstrap support (supplementary fig. 3, Supplementary Material online).

The support values of intraclade relationships were lowest within amphilophines and herichthyines. Consequently, the disagreements between the coalescent-based and the concatenated trees were also highest for these two clades (fig. 3 and supplementary fig. 4, Supplementary Material online). The amphilophines are widespread across Middle America, although their highest diversity is centered in the San Juan River and Nicaraguan Lakes (Říčan et al. 2016). We inferred two clades within the amphilophines: one clade included ((((*Amatitlania*, (*Neetroplus* + *Hypsophrys*)), *Cryptoheros*), *Chortiheros*), *Petenia*), which range from Southern Mexico to Western Panama; the other clade of amphilophines included genera distributed from the Pacific slope of northern Mexico to eastern Panama: (((*Amphilophus* + *Archocentrus*), *Darienheros*), *Mayaheros*), (*Talamancaheros* + *Isthmoheros*), that were recovered as a sister group to the genus *Parachromis*, which ranges from southeastern Mexico to western Panama.

Within the herichthyines, we recovered one major clade including the so-called “crown-group herichthyines” and a paraphyletic group of “basal herichthyine genera” (sensu Concheiro Pérez et al. 2007). The “crown-group herichthyines” included genera that extend north of the Motagua Fault to North America: *Wajpamheros*, *Theraps*, *Herichthys, Chuco*, *Cincelichthys*, *Kihnichthys, Vieja*, *Paraneetroplus*, *Maskaheros*, *Rheoheros*, and *Oscura*. *Wajpamheros* + *Theraps* were recovered as sister genera to a group formed by (*Chuco+Cincelichthys*)*, Kihnichthys*—hereafter referred as the WTC clade—, whose relationships were only recovered with moderate or low support in the ASTRAL and ML analyses (local posterior probability = 0.64, bootstrap = 67–84) (fig. 1 and supplementary fig. 1, Supplementary Material online). The aforementioned genera were recovered as the sister group to a clade in which *Vieja* was the sister genus to *Paraneetroplus* + *Maskaheros*, all of which were the closest relative of *Rheoheros* and *Oscura.* We recovered the genus *Vieja* as monophyletic in the ASTRAL and the ML trees (fig. 1 and supplementary fig. 1, Supplementary Material online), although the relationships among species showed low support and differed between the coalescent-based and concatenated trees. To the contrary, in the SVDquartets tree, *Vieja* was not monophyletic due to *Maskaheros* and *Paraneetroplus* being nested within *Vieja* (supplementary fig. 3, Supplementary Material online). *Herichthys* was recovered as the sister genus to all the other genera of “crown-group herichthyines.” Among the “basal herichthyine” genera, we found *Thorichthys* + *Trichromis* as the sister clade to the “crown-group herichthyines,” and *Chiapaheros* as the sister group to all of the above (fig. 1). This relationship was reversed in the ML and SVDquartets trees, in which *Chiapaheros* was the sister clade to the “crown-group herichthyines,” and *Thorichthys* + *Trichromis* was the sister group to *Chiapaheros* + “crown-group herichthyines” (supplementary figs. 1 and 3, Supplementary Material online). All analyses showed high support for these conflicting relationships (local posterior probability = 0.99, ML bootstrap = 100, SVDquartets bootstrap = 100).

Notably, the South American genus *Mesoheros* was recovered as the sister group to all other herichthyines; most of which are endemic to Upper Middle America and North America. Other largely non-Middle American heroines, like the South American caquetaines, were recovered as the sister clade to the amphilophines; the Greater Antillean *Nandopsis* were found to be the sister genus of amphilophines + caquetaines in the ASTRAL tree. Therefore, although our analysis lacks some of the exclusively South American lineages of heroines (e.g., mesonautines), these results agree with previous hypotheses proposing that South American and Middle American lineages of heroine cichlids are not reciprocally monophyletic, which suggests multiple colonization events of Middle America from South America (Concheiro Pérez et al. 2007; Říčan et al. 2013; Tagliacollo et al. 2017).

### Comparison of UCE and Exon Data Sets

**Fig. 2:**
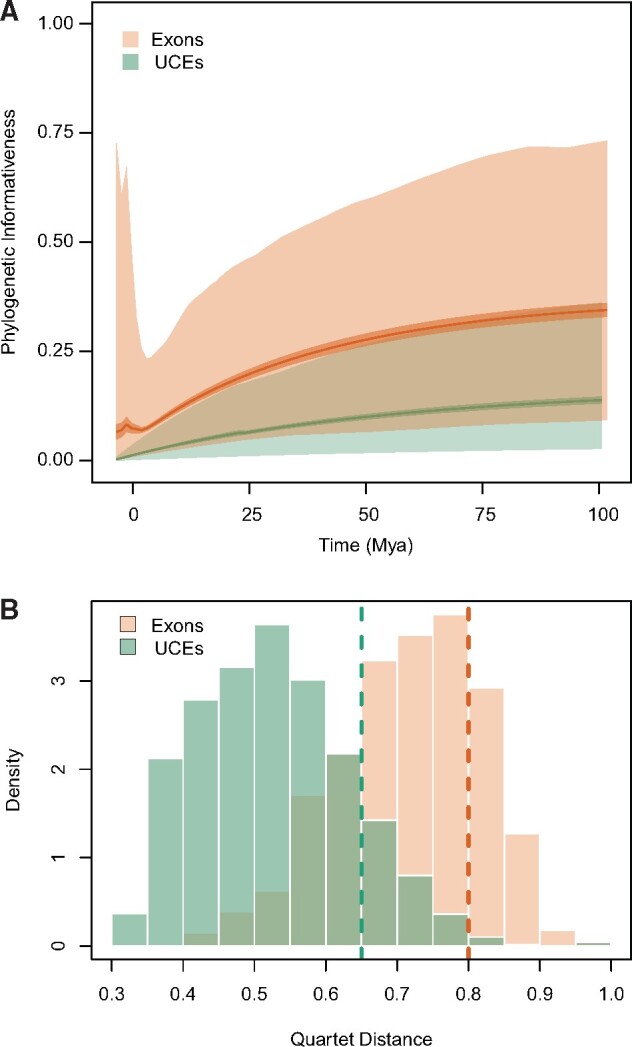
(*A*) Profiles of phylogenetic informativeness values estimated for the UCE and the exon data sets. The dark solid line is the mean value, the medium shade is 95% CI, and the light shade is the range between the 2.5th percentile and the 97.5th percentile. (*B*) Histograms showing the distribution of quartet distances among all gene trees inferred for common taxon set of cichlids the UCE and exon loci. The dashed lines indicate the average quartet distance of all gene trees to the ASTRAL species tree inferred for their respective marker type.

When comparing the common set of taxa between our UCE and the exon data set of Ilves et al. (2018) (50 species of cichlids, 45 of which are heroines; supplementary table 2, Supplementary Material online), exons were longer and contained more variable sites than the UCE alignments. Exons were on average 1,136.0 bp long and contained 118.6 parsimony-informative sites, whereas UCEs were 637.7 bp long and contained 53.2 parsimony-informative sites. Nevertheless, the relative amounts of polymorphic and parsimony-informative sites per sequenced nucleotide were similar between the two data sets: 10.5% and 23.7% versus 8.3% and 20.2%, respectively for the exons and UCEs (table 1). PI was also generally higher for exons than UCEs across most time scales, although values across loci varied and overlapped extensively (fig. 2*A*).

**Fig. 3:**
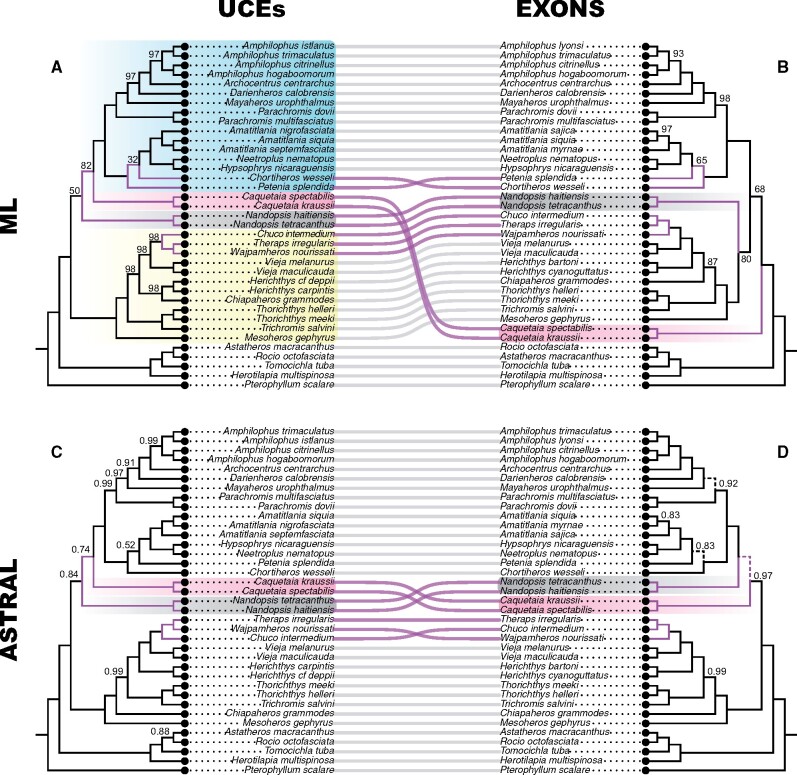
Topological comparison of the species trees inferred using RAxML and ASTRAL-III for the common cichlid taxon set and the UCE (*A* and *C*) and exon (*B* and *D*) data sets. All nodes are supported by bootstrap values=100, or local posterior probabilities=1.0, unless otherwise noted. In (*D*), dashed lines indicate internodes that are potentially in the anomaly zone because they were shorter than the limit of *a(x)*. Heroine groups that differ in their relative phylogenetic position among analyses are highlighted in blue (amphilophines), pink (caquetaines), gray (*Nandopsis*), and yellow (herychthyines).

The phylogenetic hypotheses generated from the full list of taxa in the exon (Ilves et al. 2018) and UCE data sets each recovered identical topologies to their respective exon and UCE common taxon sets that shared the same species. The only exception was the UCE ML tree that showed virtually no support for the placement of *Nandopsis* (fig. 3, bootstrap = 50). This consistency across taxon sets suggests that, in our case, reducing the taxon sampling in the common taxon set did not affect the topology of the trees inferred. The UCE and the exon-based trees were also highly congruent with each other, although a few clades recurrently disagreed, namely the relationships among the major clades of Heroini and within the most recent divergences of the WTC clade of herichthyine genera (fig. 3 and supplementary fig. 5, Supplementary Material online).

The most significant differences among analyses were those related to the relationships among the major clades of heroine cichlids with the greatest biogeographic implications. For example, whether the Middle American amphilophines were more closely related to the South American caquetaines or the Greater Antillean *Nandopsis*, and the relative position of these lineages to the other major Middle American lineage of herichthyines can severely influence historical biogeographic estimation. In general, UCEs supported the amphilophines + *Caquetaia* hypothesis and exons supported the hypothesis of amphilophines + *Nandopsis* (fig. 3).

Some specific differences that we observed between tree topologies were due to the type of marker (e.g., exons or UCEs) and these differences were independent of the inference method used. For example, within herichthyines, concatenation and coalescent-based analysis of the UCE data always recovered *Wajpamheros+Theraps* as the sister group to *Chuco*, whereas all trees inferred using the exon data recovered *Wajpamheros* as the sister group to *Theraps+Chuco* (fig. 3). At a deeper scale, all UCE-based trees recovered a sister relationship between the Middle American amphilophines and South American caquetaines; however, exons recovered a different relationship among these groups in every phylogenetic analysis but never amphilophines + caquetaines.

Other differences that we observed among phylogenetic trees were associated with the inference method and were independent of the marker type. Among the main clades of heroine cichlids, analyses in concatenation of both UCE and exon loci recovered herichthyines as the sister group to *Nandopsis*, and all the coalescent-based species trees recovered a monophyletic amphilophines + *Nandopsis* + caquetaines (although there were also differences in the relationships within that latter assemblage; fig. 3 and supplementary fig. 8, Supplementary Material online). As noted earlier, the ML tree of the UCE common taxon set was the only one where the relationships of *Nandopsis* were poorly supported (bootstrap = 50, fig. 3).

Heterogeneity estimates based on mean quartet divergences were virtually identical between individual gene trees and species trees inferred using a concatenation or a coalescent approach. The exon-based species trees shared ∼80% of their quartets with the average exon gene tree, whereas the UCE species trees shared, on average, 65% of their quartets with individual UCE gene trees. Overall, the average percentage of shared quartets among all exon gene trees was 71.6% (SD = 10.1), and 52.8% (SD = 10.7) among all UCE gene trees (fig. 2*B*).

Gene concordance (gCF) among exon gene trees was higher than for UCE gene trees: average exon-gCF = 47.2 (SD = 27.0) and UCE-gCF = 31.6 (SD = 21.9). Conversely, site concordance (sCF) was higher for UCEs than for exons, although this difference was nonsignificant: average exon-sCF = 62.7 (SD = 18.2) and UCE-sCF = 64.9 (SD = 18.1) (*t*-test, *t*(92) = −0.591, *P *=* *0.556). For both sets of marker types, we observed a wide range of gCF and sCF values, and in most branches sCF values were larger than gCF values. This pattern was more evident for the UCE data, for which only two branches showed lower sCF than gCF values; the shorter length of the average UCE loci may be the reason for the lower gCF values in UCEs versus exons (supplementary fig. 6, Supplementary Material online).

Among the 48 branches in the “common taxon set” species trees, the UCE-based tree had 10 branches where discordance deviated significantly from a pure ILS model (i.e., equal frequencies of gene trees or sites for the alternative topologies): two branches where the number of gene trees supporting the alternative topologies was significantly different (χ^2^, *P *<* *0.05), five where the number of sites were significantly different, and four where the number of gene trees and sites differed significantly. In the exon-based tree, 15 branches showed significant differences in the number of gene trees or sites supporting the alternative topologies: in two branches the number of gene trees was significantly different, in one branch the number of sites was significantly different, and in 12 branches both gene trees and sites were significantly different.

**Fig. 4:**
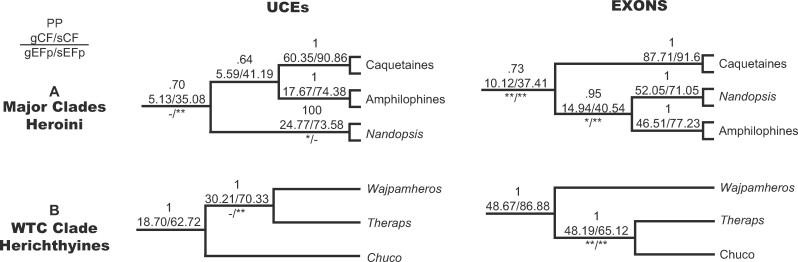
Cladograms representing conflicting relationships of heroine cichlids between the UCE and the exon data sets. Values above each branch indicate local posterior probabilities (PP) from the ASTRAL species trees and the gene concordance (gCF) and site concordance values (sCF). Below the branches, we indicate whether there is a significant probability that the data can reject equal frequencies of gene trees (gEFp) or sites (sEFp) supporting the alternative topologies.

For the relationships among the major clades of heroine cichlids (amphilophines, caquetaines, and *Nandopsis*), UCEs showed lower gCF values in both branches of the most frequent topology (gCF = 5.13–5.59) than the exons (gCF = 10.12–14.94) but similar sCF values (UCE sCF = 35.08–41.49, exons sCF = 37.41–40.54) (fig. 4*A*). We observed more similar sCF between topologies for the deepest branch of this lineage, whereas in the more terminal branches, sCF were much larger for the most frequent topology than for the alternates (supplementary fig. 7, Supplementary Material online). In the exon-based tree, two branches of this clade had significantly different frequencies of gene trees and sites that supported the alternative topologies, whereas in the UCE tree only the deepest node of this clade showed a significant difference in the number of sites supporting alternative topologies (fig. 4 and supplementary fig. 7, Supplementary Material online). This pattern is congruent with a larger effect of ILS at older divergence times for the UCE data than for the exons. In the WTC clade of Upper Middle American herichthyines, gCF values were lower for the UCEs than in the exon species tree (UCE gCF = 30.21, exons sCF = 48.19) and sCF were higher for the UCEs than for the exons (UCE sCF = 70.33, exons sCF = 65.12) (fig. 4*B*). In this case, all the concordance factors for the branches in the most frequent topology were much higher than for the less frequent ones (supplementary fig. 7, Supplementary Material online). One branch in the exon-based tree significantly rejected the ILS hypothesis of equal frequencies for both gene trees and sites supporting alternative topologies but in the UCE tree, that same branch only rejected the ILS hypothesis for the number of sites (fig. 4 and supplementary fig. 7, Supplementary Material online).

**Fig. 5:**
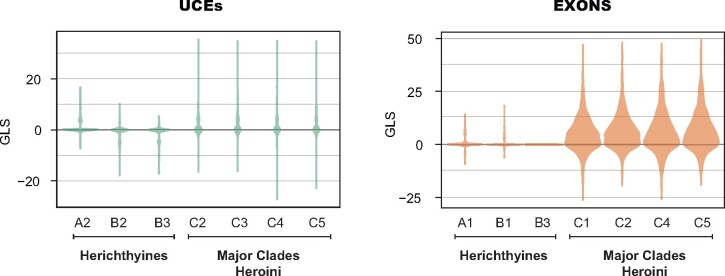
Violin plots showing the distribution of ΔGLS values (following Shen et al. 2017) across gene trees for each topology test and genomic marker type.

In the topology tests, we observed a general pattern for both types of markers in which most loci had low support for either of the topologies tested with average values ranging between 1.764 x 10^−6^ and 6.462 for the exons, and between −1.192 and 2.404 for UCEs (fig. 5). Interestingly, both types of markers agreed on the comparisons with the highest and lowest average ΔGLS values (fig. 5 and supplementary table 3, Supplementary Material online). In most comparisons, the majority of loci supported the unconstrained topology, and longer loci with more informative sites also had more power for driving the unconstrained topology, although this relationship was not always positive (e.g., UCE comparisons of herichthyines B1 vs. B2 and B1 vs. B3) or significant (e.g., UCE comparisons of herichthyines A1 vs. A2, and exon comparisons of herichthyines A2 vs. A1, B2 vs. B1, and B2 vs. B3, supplementary fig. 9 and table 3, Supplementary Material online).

More exons than UCEs showed very strong support toward one particular hypothesis. Across the seven topologies tested, 20 exonic loci recurrently showed ΔGLS values greater than or equal to 25. As a reference, among all the branches and genes included in two animal and plant data sets used in the original description of this method (Shen et al. 2017), only 4–6% of loci showed ΔGLS values ≥25, >96% of which consisted of control branches or noncontentious relationships such as the monophyly of amniotes, mammals, or seed plants (Shen et al. 2017). Conversely, only three UCE loci showed ΔGLS ≥25 (supplementary table 3, Supplementary Material online). When we pruned these “strongly informative” loci from their respective data sets and reinferred the species trees as described above, the UCE-based ML phylogenetic hypothesis recovered was identical to the unpruned data set but for the exon data set, the pruned ML concatenated tree recovered *Nandopsis* as the sister group to amphilophines instead of as the sister group to herichthyines [((amphilophines, *Nandopsis*), herichthyines), caquetaines vs. ((herichthyines, *Nandopsis*), amphilophines), caquetaines] (supplementary fig. 10, Supplementary Material online). The species trees inferred in ASTRAL were identical between the pruned and unpruned data sets for both types of genomic markers.

In the exon species tree, we found three internodes that potentially are in the anomaly zone. All the internodes that were shorter than the limit of the anomaly zone, *a(x)*, were in the clade including caquetaines, *Nandopsis*, and amphilophines: The internode between the common ancestor of caquetaines and *Nandopsis* + amphilophines, and two internodes at the base of the each of the subclades of amphilophines (fig. 3). Conversely, we did not find nodes in the anomaly zone in the UCE tree.

The ABBA-BABA tests only showed one pattern suggestive of hybridization among the major clades of Heroini in the UCE species tree. In this case, the significantly negative *D*-statistic indicates an excess of BABA pattern trees which is compatible with gene flow between amphilophines and *Nandopsis*. In the exon species tree, the *D*-statistic was also negative but not significant. In the case of the WTC clade, the *D*-statistics estimated for UCEs and exons were positive and not significant (table 2).

**Table 2:** Results of the ABBA-BABA Tests for the Two Discordant Clades and Type of Markers. Significant *D*-Statistics (*Z* Score>3 and *P* < 0.0001) are Highlighted in Bold.

Clade	Marker	P1	P2	P3	*D*-Statistic	*Z* score
WTC Clade Herichthyines	UCEs	*Wajpamheros*	*Theraps*	*Chuco*	0.33121	1.6017
	Exons	*Theraps*	*Chuco*	*Wajpamheros*	0.00453	0.0339
Major Clades Heroini	UCEs	Amphilophines	Caquetaines	*Nandopsis*	−0.807	**−14.2104**
	Exons	Amphilophines	*Nandopsis*	Caquetaines	−0.0696	−1.7727

## Discussion

Marker selection is an important, although often overlooked, component in the design of phylogenomic studies (Gilbert et al. 2015). Our study confirmed the robustness of phylogenomic inference to the use of marker types with different biological and phylogenetic properties. Interestingly, the few contentious relationships in the phylogeny of Middle American cichlids disagreed between types of genomic markers and also based on inference methods; this result suggests common pitfalls for both types of markers. The regions of disagreement that we explored had in common generally low phylogenetic signal that was overwhelmed by ILS or nonphylogenetic signals. ILS was more prevalent in the UCEs (particularly in the older contentious relationships), whereas potential systematic errors were detected in the exon data set. Our study corroborates the general finding that despite the great promise of next-generation sequencing technology, hard phylogenetic problems remain a challenge in evolutionary biology.

### Evolutionary Relationships of Neotropical Heroine Cichlids

Much progress has been made in recent years in our understanding of the evolutionary relationships among Neotropical cichlids, a group of fishes with historically enigmatic phylogenetic hurdles at various taxonomic scales (McMahan et al. 2013; Arbour and López-Fernández 2016; Matschiner et al. 2020). Although the complete exon and UCE data sets differ considerably in taxon sampling for some clades (e.g., *Australoheros*, *Mesonauta*), and particularly at shallow taxonomic or intrageneric scales, the overall relationships recovered are largely congruent (Ilves et al. 2018). Across heroines, the major incongruence is the phylogenetic position of the Greater Antilles endemic *Nandopsis*. This clade of cichlids has long been of interest to biogeographers given its potential to offer insight into the evolutionary history of colonization by freshwater fishes on the Caribbean islands (Chakrabarty 2006; Tagliacollo et al. 2017). Based on analyses of our novel UCE data, incongruence in the position of *Nandopsis* differs by inference methods, with coalescent analysis supporting a sister relationship with amphilophines + caquetaines (fig. 1), and ML analysis supporting a sister relationship with herichthyines (supplementary fig. 1, Supplementary Material online). Phylogenetic hypotheses based on exons (Ilves et al. 2018) are in agreement with our UCE results, supporting the hypothesis that taxon sampling and the type of genomic marker used likely do not explain incongruence in the position of *Nandopsis*. Our comparative analyses of a common taxon data set for UCEs and exons show the same results with exception of the UCE ML analysis, where *Nandopsis* switches to a relationship with amphilophines + caquetaines; however, this phylogenetic arrangement is not well supported (fig. 3*A*). In this case, taxon sampling may play a role in phylogenetic reconstruction since with the full UCE data set there is considerably more support for the position of *Nandopsis*.

Conclusions drawn regarding the biogeographic and evolutionary history of the Greater Antilles will be influenced by the phylogenetic position of *Nandopsis*. These are the only major lineage of cichlid fishes not naturally found on (or connected to) a former Gondwanan landmass and their presence on these islands can best be explained by phylogenetic support of alternative vicariance scenarios or oceanic dispersal (Chakrabarty 2004). As explained by Ilves et al. (2018), studies often base these conclusions on a single phylogenetic reconstruction. Even if the data set is novel, larger, or more taxonomically complete, well-documented cases of incongruence hinder the robustness and accuracy of historical reconstructions. Furthermore, recent discussions have highlighted the fact that nodal support measures, such as nonparametric bootstrap, increase with the size of the data set and can give a false sense of “confidence” in phylogenetic relationships (Liu et al. 2015; Simon 2020). Therefore, it is necessary to interrogate genomic data sets and explore alternative measures of support to ensure a more rigorous evaluation and a more robust interpretation of the data (Arcila et al. 2021). The *Nandopsis* clade is a phylogenetically difficult lineage to unambiguously resolve, and we are unable to do so comparing two independent and large genomic data sets with robust taxon sampling and varied phylogenetic analyses. New data and novel analysis types are important to continue to pursue; however, we agree with Ilves et al. (2018) that alternative phylogenetic hypotheses should be concurrently evaluated when studying the evolutionary history of Middle American cichlids.

Another incongruence concerns the phylogenetic position of the upper Río Grijalva endemic cichlid *Chiapaheros grammodes*, a monotypic genus from Guatemala and Mexico (Elías et al. 2020). Data sets for UCEs and exons differ in taxon sampling but overall relationships within the herichthyines are largely the same except for those of *Chiapaheros*. Coalescent-based inference recovers *Chiapaheros* as the sister genus to all herichthyines minus *Mesoheros* (and presumably *Chocoheros*, which was not sampled), whereas concatenation recovers *Chiapaheros* as the sister group to all herichthyines minus *Thorichthys*, *Trichromis*, and *Mesoheros* (in the ML UCE tree with all taxa; supplementary figs. 1 and 2, Supplementary Material online). Although this result supports an analytical explanation for the incongruence, it is notable that the position of *Chiapaheros* in the concatenated ML tree of Říčan et al. (2016) based on ddRAD sequencing data matches the coalescent-based results for UCE and exon markers. Thus, it is possible the explanation is not solely analytical and the difference could also be based on the type of data (i.e., marker choice). Unfortunately, at the time we analyzed the UCE and exon datasets for this study, the data from Říčan et al. (2016) were not yet publicly available, thus preventing the ability to further test these results. Future studies that compare all available datasets are warranted. Sequence data generated for phylogenetic and other studies have long been deposited in public repositories such as GenBank and their accession numbers made available in the original publication. Importantly, this readily allows access for replication of analyses and corroboration of results. In this context, genomic data are no different than Sanger data (i.e., individual loci or genes) and scientific journals should be responsible and require all authors publish the source of their genetic and specimen data concurrently with their results (Colella et al. 2020; Buckner et al. 2021).

The genus-level taxon sampling for our heroine cichlids most closely matches that of Říčan et al.’s (2016) phylogenetic study using ddRADs. Figure 5 of that paper depicts relationships but with a focus on the discordance between markers; however, the examination of this phylogeny alone could be misleading given that some species in their figure are lacking genomic data (e.g., *Kihnichthys ufermanni*, discussed below). Thus, our comparisons with Říčan et al. (2016) are based on the ML analysis of ddRAD data in the supplementary files of that study. In addition to the incongruence concerning *Chiapaheros* discussed above, there are other genus-level differences. Říčan et al. (2016) make the claim that the monotypic *K. ufermanni* is likely congeneric with *Cincelichthys* based on shared tooth morphology. Artigas Azas (2020) later proposed the synonymy of these two genera based on observations of similarities and misinterpretation of supposed thresholds for measures of phylogenetic support (e.g., Říčan et al. 2016 did not have genomic data for *Kihnichthys* in their study). Our UCE phylogeny includes *Kihnichthys* and both species of *Cincelichthys*, and we recovered *Kihnichthys* as the sister group to *Cincelichthys* + *Chuco* in both concatenation and coalescent inferences (fig. 3). Our results disagree with the conclusions of Artigas Azas (2020) and support the current validity of these two genera pending future work and scrutiny of phylogenetic data. Additionally, the ddRAD phylogeny of Říčan et al. (2016) recovers the monotypic *Oscura heterospila* as part of the *Vieja* clade (sensu McMahan et al. 2015). However, Říčan et al. (2016) note this was based on a single sample from a juvenile specimen. We extracted the complete cytochrome *b* and cytochrome oxidase *I* mitochondrial genes from the UCE raw data of our sample of an adult *O*. *heterospila* (LSUMZ 16229) (GenBank accession numbers MW365944 and MW365943, respectively). Both gene sequences blasted with 100% similarity to sequences from individuals of *O*. *heterospila* on GenBank (i.e., AY843414, HQ424213, and GU817280). Thus, we regard the individual in Říčan et al. (2016) as a likely misidentification, leaving the genus *Oscura* absent from their ddRAD data set. This highlights the importance of discoverable and accessible voucher specimens linked to sequences to permit the substantiation of identifications (Chakrabarty et al. 2013).

### Differences between Genomic Marker Types and Inference Methods

We compared the genetic variability and PI of exon and UCE loci. On average, exons were longer, had approximately double the number of polymorphic sites, and were more informative than UCEs. This outcome contradicts previous comparative studies and the expectation that exons have a slower evolutionary rate than the flanking regions of UCEs (Gilbert et al. 2015; Reddy et al. 2017). Despite this, the percentage of polymorphic or informative sites per nucleotide was similar for both types of markers, although still smaller for UCEs. These values match the general observation that locus length is an important predictor of genetic variation and phylogenetic information, although not the only one (Kuang et al. 2018; Alda et al. 2019). We also observed a generally positive relationship between locus length and their strength of support (ΔGLS) for most of the topologies tested (supplementary fig. 9, Supplementary Material online). Despite being generally small or nonsignificant, the effect sizes of the linear regressions were always larger for UCEs than for exons, which may suggest that the longest exon loci might not necessarily be the ones containing the most informative sites and/or may contain more noise (see below).

The differences in length between the two marker types could be due, first, to the fact that exon hybridization was carried out with more probes per locus (Ilves and López-Fernández 2014), which allowed sequencing and assembling longer contigs, whereas UCEs were hybridized with probes that only target the conserved core region (Faircloth et al. 2012). Second, we carried out internal trimming of our UCE alignment to remove poorly aligned regions, as opposed to the exon study (Ilves et al. 2018). Excluding poorly aligned sites, however, comes at the cost of also reducing phylogenetic signal (Fan et al. 2020).

Another major difference between genomic marker types was the greater gene tree heterogeneity and gene tree-species tree heterogeneity of UCEs compared with exons (fig. 2*B*), a finding that has also been highlighted in other comparative studies (Arcila et al. 2021). This result is not unexpected if we assume that the shorter and less informative UCEs will produce more inaccurate gene trees than the exons (Camargo et al. 2012). In addition to stochastic or systematic errors, heterogeneity could also be due to higher ILS of UCEs. It has been proposed that stronger selective pressure over coding sequences may reduce their effective population size and in consequence, their coalescence time (Scally et al. 2012). Our results suggest a larger role of ILS on UCE gene tree heterogeneity. Only one branch in the “common taxon set” tree showed a significant difference between gene tree frequencies supporting the alternative topologies, that is, deviating from the null hypothesis of ILS as the only source of gene tree discordance (supplementary fig. 7, Supplementary Material online).

In a scenario of genomic marker types with varying levels of discordance, we expect that inference methods will provide more accurate estimates of the true species tree depending on their capability for accommodating gene tree heterogeneity. This difference in performance alone could explain the differences observed between marker types and between inference methods (Ilves et al. 2018). Therefore, for UCEs we favor the ASTRAL species tree, which performs best for short genes and high ILS, over the concatenated ML tree and SVDquartets tree (whose accuracy is only comparable to ASTRAL’s under conditions of low ILS; Chou et al. 2015). Similarly, one could argue that the exon ML concatenated tree is more accurate than the UCE tree; however, we found additional evidence suggesting that factors other than ILS might be causing exon gene tree discordances. For example, and in contrast to UCEs, there were three branches in the exon species tree with significant differences in the frequency of gene trees supporting the two discordant topologies. For one of these branches, the most common gene tree topology is not the same we recovered in the ML concatenated tree (ML tree gCF = 10.12 vs. alternative quartet 2 gCF = 16.63, supplementary fig. 7, Supplementary Material online), a pattern expected if the branch is in the anomaly zone (Degnan and Rosenberg 2006). Notably, we also identified this same branch at the stem of (amphilophines + *Nandopsis*), caquetaines as having an internode length shorter than the theoretical limit of the anomaly zone (fig. 3*D*). The presence of anomalous exon gene trees might be responsible for the incongruence between genomic marker types—we did not find evidence of anomalous UCE gene trees—, and between inference methods because the ML tree may favor the anomalous tree topology (Kubatko and Degnan 2007).

### Support for Contentious Relationships

The relationships among amphilophines, caquetaines, and *Nandopsis* were one of the major sources of disagreement in ours and previous studies (Ilves et al. 2018). The topology tests involving these clades revealed differences and commonalities between types of genomic markers. In agreement with the higher PI and number of informative sites of exons, we found that this marker type also had overall higher levels of support (ΔGLS) for any of the topologies tested. Exons also showed more loci than UCEs with extremely high support values (20 exon loci vs. three UCE loci with ΔGLS values ≥25), and this small number of loci may be exerting a disproportionate amount of influence on the resolution of contentious branches (Fong et al. 2012; Arcila et al. 2017; Shen et al. 2017). When loci with ΔGLS ≥25 were removed from their respective data sets, we observed that the pruned exon ML topology differed from the original tree, but not when we removed 20 random loci, or in either of the coalescent-based species trees. Most importantly, the discordant nodes between the pruned and nonpruned exon ML concatenated trees were again those involving the relative position of the main clades of heroine cichlids (supplementary fig. 10, Supplementary Material online), which supports the strong influence that a few loci have on the resolution of these internodes.

Not only were highly informative loci found for the relationships among the main clades of heroines, but the average ΔGLS of all exons was much larger for these comparisons than in any of the WTC clade. The average ΔGLS values of the WTC clade comparisons were only slightly larger than 0, indicating very little to no support for either of the topologies tested (supplementary table 3, Supplementary Material online). This pattern is the opposite to what we would expect from the concordant factor analysis, where gCFs and sCFs were much larger for the WTC clade branches (minimum gCF: 10.12% and sCF: 37.41% compared with minimum gCF: 48.67% and sCF: 65.12%; supplementary fig. 7, Supplementary Material online). However, a closer examination to the data revealed that fewer decisive sites are informing the branches of the WTC clade than among the main heroine clades. As a result, and despite the larger sCFs, the total number of concordant sites in the WTC clade was much smaller—that is, there was a minimum of 65 concordant sites in the WTC branches compared with a minimum of 315 concordant sites in the branches of the heroine clade (supplementary fig. 7, Supplementary Material online). This observation is important because first, it serves as a warning for the use of bootstrapping as a measure of support for conflicting relationships (Salichos and Rokas 2013), since a very small number of sites can still produce branches with high support (all nodes are supported by bootstrap values = 100, or local posterior probabilities = 1.0; fig. 3), and second, it highlights the complementary information provided by these statistics. For example, the calculation of ΔGLS is based on the average difference of log-likelihood values between two tree topologies across all sites within a locus (Shen et al. 2017), whereas sCFs rely on the percentage of decisive sites that support a particular tree branch regardless of where they occur (Minh, Hahn, et al. 2020). Therefore, a nonrandom distribution of informative sites will have a more significant impact on ΔGLS than on sCFs. Similarly, a small number of very large ΔSLS values may bias a locus’ ΔGLS but it will not affect the sCF. We found evidence for either or both of these factors playing a role in the observed discrepancies, as revealed by the nonsignificant or not positive correlation between locus length and ΔGLS for the topology tests of the WTC clade (i.e., loci with more sites were not necessarily more informative about these branches); whereas the correlation was always positive for the comparisons among the main clades of heroines (supplementary fig. 9, Supplementary Material online).

### Phylogenetic Signal versus Noise and Error

The highly informative loci in the exon data set may be related to their evolutionary rates being accelerated or shaped by positive selection which can mislead phylogenetic inference (Yang 1998), and/or to systematic errors. For example, misspecified evolutionary models may have prevented the accurate reconstruction of the evolutionary history of these genes (Phillips et al. 2004). Both of these nonmutually exclusive explanations are in agreement with our observations. First, we expect that exons will be under stronger positive selection than noncoding UCEs, and second, modeling the evolution of coding sequences has also proven more complicated than noncoding sequences. Therefore, many of the disagreements between data sets may be a reflection of model violations in our phylogenetic analyses (Reddy et al. 2017). In our case, we observed that exons have a higher GC content (exon GC frequency mean = 0.5, SD = 0.04 vs. UCE GC frequency mean = 0.4, SD = 0.05) and significantly heterogeneous base composition (χ^2^ test of homogeneity of state frequencies across taxa: χ^2^ = 662.092 [147, *N *=* *50], *P *<* *0.001) in contrast to UCEs (χ^2^ = 145.53 [147, *N *=* *50], *P *=* *0.519), all of which could be causing biased estimates. Nevertheless, UCEs are also under selection (Katzman et al. 2007) and yet another possibility for the differences observed between marker types is that internal trimming with Gblocks had removed the most divergent sequences from the UCE alignments. As we mentioned earlier, removing poorly aligned regions may result in a loss of information by reducing the influence of outlier genes (Fan et al. 2020), like those with very large ΔGLS or PI. As a result of trimming, poorly aligned regions that might represent highly divergent or rapidly evolving sites are removed which can have a similar impact as other data filtering strategies that decrease nonclocklike sites and base compositional heterogeneity (Kuang et al. 2018; Alda et al. 2019); that is, the data may be less “messy” but also potentially less informative when these poorly aligned regions are removed.

As our data processing takes a conservative approach, we may argue that the gene tree/species tree discrepancies in UCEs are largely due to high ILS rather than to systematic errors. On the other hand, the phylogenetic signal of UCEs seems to result from the cumulative addition of poorly informative loci that disagree at random, instead of being driven by a small number of discordant and/or highly informative loci. However, other processes could also be the cause for disagreement and be confounded for ILS. It has been shown that hybridization is a major driver for the adaptive radiation of cichlids (Seehausen 2004; Irisarri et al. 2018), but if gene flow occurs during speciation it cannot be discerned from ILS and will add to the effect and duration of ILS (Suh 2016); however, a signal of postspeciation gene flow was only detected in the UCE data set. The significant *D*-statistic suggests that gene tree discordance could be due to gene flow between amphilophines and *Nandopsis*, which is also compatible with the extensive mitonuclear discordance observed among the major clades of Heroini (Říčan et al. 2016; and references therein). Additionally, it is remarkable that exons did not show a significant signal of introgression. Under a neutral model, a pattern of introgression should be equally prevalent across the genome and therefore detectable by any type of marker (Durand et al. 2011). However, gene flow between species is heterogeneous and may be limited by hybrid incompatibility or reduced recombination rates of regions under strong selection (Wu 2001; Martin et al. 2015). The exon loci that we analyzed are likely under varying levels of selection, and some of them have played a prominent role in the rapid adaption and radiation of cichlid fishes following their colonization of Middle America (Hauser et al. 2017; Ilves et al. 2018). Nevertheless, our data cannot discern whether the differences between genomic regions are due to a prominent role of exons in speciation, and/or to species boundaries that are more porous to the introgression of UCEs due to lower selective pressure.

## Conclusion

Overall, our results underline the intricacies of phylogenomic data, how relationships can be driven by a handful of sites or loci, and how the support on these branches may vary depending on the distribution of informative sites across the genome. Although our work focused on Neotropical cichlids, the comparisons made here can help target strategies for refining other difficult questions across the Tree of Life and highlight the importance of exploring the causes of discordant results between different analyses and different phylogenomic data sets.

In our study, despite differences in their informativeness and gene tree discordance, UCEs and exons overwhelmingly agreed on most of the relationships resolved, and also agreed on the few contentious nodes that both types of markers failed to resolve consistently.

Based on the differences observed, studies using UCEs may require more and longer loci to account for the larger gene tree heterogeneity. However, because this variation is mostly due to ILS, these markers are more suitable for being analyzed using coalescent methods that can accommodate this type of random heterogeneity. Exons, being more informative as a unit, might be more suitable to resolve ancient relationships. On the other hand, if exons are more prone to show loci with increased rates or that deviate from generalized evolutionary models, we recommend exploring the relative support of loci for some relationships and filter those that have an overwhelming effect on the topologies. It must also be considered that a single tree most likely will not be capable of encapsulating the complex evolutionary histories of many organisms. Therefore, we agree with previous authors proposing that comparative studies should be based on multiple trees—inferred using different methods and/or molecular markers—to account for uncertainties in the Tree of Life (Arcila et al. 2021).

Given the very low phylogenetic signal shown by all markers, it seems that unless future studies are capable of dramatically increasing the amount of sequence data or the accuracy of evolutionary models, the relationships at the root of heroine cichlids will not be satisfactorily resolved. Finally, for this and any other study, we propose that following the exploration and detection of highly conflicting relationships, the use of whole-genome sequencing data should be focused on targeting markers with enough potential phylogenetic signal to resolve problematic regions. Future genomic work might identify new regions of the genome that have desirable properties related to phylogenetic signal and functionality that could be targeted as molecular markers in nonmodel organisms and improve species tree reconstruction (Van Dam et al. 2021). At the present, understanding the biological complexity of currently available molecular marker types and incorporating these features into realistic evolutionary models will be necessary to overcome differences in limitations of phylogenomic analyses (Reddy et al. 2017; Simion et al. 2020).

## Materials and Methods

### Taxonomic Sampling

Our taxon sampling is composed of 93 individuals from 88 species of cichlid fishes. The ingroup includes data from 72 species spanning 39 genera of cichlids in the tribe Heroini, representing all major clades within this tribe. We also included 15 species as outgroups: two species from the Neotropical tribe Cichlasomatini, eight species of Geophagini, and five species of the African subfamily Pseudocrenilabrinae. We used one sample of *Etroplus suratensis* from the Indian subcontinent and the subfamily Etroplinae to root the tree. Samples were collected by the authors, loaned from natural history collections, or data mined from online repositories (supplementary table 1, Supplementary Material online). Species nomenclature follows the most recent taxonomy available in the Eschmeyer Catalog of Fishes (Fricke et al. 2021).

### Laboratory Methods

We extracted DNA from muscle or fin tissue using the QIAGEN DNeasy kit (Qiagen). We used approximately 500 ng of DNA as starting material to construct dual-indexed (Faircloth and Glenn 2012) genomic libraries using the Kapa Hyper Prep Kit (Kapa Biosystems) that we pooled and enriched following a target hybrid capture approach (http://ultraconserved.org) with slight modifications (Burress et al. 2018). We used the MYbaits UCE Actinopterygians 0.5Kv1 or Acanthomorph 1Kv1 capture kits (Arbor Biosciences) that respectively target 500 and 1,000 UCE loci across ray-finned fishes (Faircloth et al. 2013; McGee et al. 2016), and sequenced all enriched libraries in several lanes of PE150 Illumina HiSeq 3300 at the Oklahoma Medical Research Foundation (OMRF).

We additionally extracted UCEs in silico from four genome-enabled species using available scripts (http://github.oliveros.git) and from raw sequencing data of six outgroup species from previous publications (Burress et al. 2018) uploaded to the Sequence Read Archive (SRA) (supplementary table 1, Supplementary Material online). Our data are available at the SRA (BioProject P RJNA690533) and the Dryad Digital Repository (Alda et al. 2021, see Data Availability statement below).

### Processing and Phylogenetic Analysis of UCEs

We trimmed demultiplexed sequences to remove adapters and low-quality bases using default settings in trimmomatic (Bolger et al. 2014) and assembled the trimmed FASTQ data into contigs using SPAdes (Bankevich et al. 2012) and the PHYLUCE package (Faircloth 2016). We used additional scripts within PHYLUCE to: identify UCE loci from assembled contigs, align UCE loci using MAFFT (Katoh and Standley 2013), trim internal poorly aligned regions with Gblocks (Castresana 2000), compute alignment statistics, and prepare alignments for phylogenetic analysis. We used these steps to create alignments that were at least 75% complete (i.e., a minimum of 70 out of 93 samples were present in each locus alignment).

We concatenated all individual loci to infer a ML phylogenetic hypothesis using RAxML v.8.0.19 (Stamatakis 2014). We considered each locus as an independent partition under the GTRGAMMA substitution model and conducted 40 ML searches for the phylogenetic tree that best fit the data. Following the search for the best tree, we used RAxML to generate nonparametric bootstrap replicates using the autoMRE option, and we reconciled the best-fitting ML tree with the bootstrap replicates using RAxML.

To account for gene tree incongruences and coalescent stochasticity among individual UCE loci, we inferred a species tree using the summary coalescent-based method of ASTRAL-III v.5.7.3 (Zhang et al. 2018). ASTRAL-III uses a quartet-based approach to find the species tree that shares the maximum number of quartets within a set of gene trees. For this purpose, we first used RAxML to infer individual gene trees and assessed their nodal support creating 200 bootstrap replicates for each UCE locus. Then, we used these gene trees as input for ASTRAL and assessed branch support of the species tree using local posterior probabilities (Sayyari and Mirarab 2016). ASTRAL is a summary method, where species tree inferences rely on accurately estimated gene trees and are therefore sensitive to poorly supported branches (Zhang et al. 2018). As suggested by Zhang et al. (2017), we repeated the species tree analysis twice using the same set of input gene trees after removing branches with very low support—that is, after collapsing all nodes with bootstrap ≤1 and with bootstrap ≤10 using Newick Utilities 1.6 (Junier et al. 2010).

We also constructed a species tree using SVDquartets (Chifman and Kubatko 2014) as implemented in PAUP v.4.0a150 (Swofford 2003). The SVDquartet method does not rely on a prior inference of individual gene trees; rather, it uses single-site patterns to estimate the species tree in a way that is statistically consistent with the multispecies coalescent. The algorithm uses multilocus SNP data to infer quartet trees for subsets of four species in a coalescent framework and then combines the set of quartet trees into a species tree using a supertree method (Chifman and Kubatko 2014). We evaluated 100,000 random quartets and performed 1,000 bootstrap replicates of the data to assess support and then assembled the species tree using the quartet max-cut method (Snir and Rao 2012).

### Comparing Phylogenomic Analyses of UCEs and Exons

We compared the phylogenomic hypotheses proposed by our newly generated UCE data to the exon-based hypotheses from Ilves et al. (2018). The original exon data set from this study includes 139 species of cichlids, of which 128 were Neotropical species and 57 were heroine cichlids that were sequenced for 415 exon loci (Ilves et al. 2018). For accurately comparing the two data sets, we pruned them to create a common taxon set that includes the same 50 species (45 ingroup and five outgroup species). In the few instances where the same species were not found between data sets (five in the ingroup and nine in the outgroup), we used the most closely related species from the same genus that we had available and that unequivocally belong to the same lineage (supplementary tables 1 and 2, Supplementary Material online). Then, we reanalyzed the common taxon sets following the same methodology as explained above: ML tree inference using RAxML of the concatenated data partitioned by locus, and inference of coalescent-based species trees using ASTRAL-III and SVDquartets.

We computed and compared summary statistics including locus length and the number of polymorphic and parsimony-informative sites for each locus and genomic marker type using scripts in the PHYLUCE package (e.g., *phyluce_assembly_get_fasta_lengths*, *phyluce_align_get_align_summary_data*, *phyluce_align_get_informative_sites*). We also calculated PI for each locus using the web application PhyDesign (López-Giráldez and Townsend 2011). PI estimates the probability of a locus to resolve a given node in the tree and the shape of its profiles can provide information about the utility of molecular sequences for inferring evolutionary relationships at specific time points (Townsend 2007; Dornburg et al. 2017). Before estimating PI we created timetrees that were used as input in the program PhyDesign. We transformed our best-fit ML trees for the common taxon sets of UCEs and exons into time-calibrated trees using the penalized likelihood method and the noncorrelated rates of molecular substitution (“relaxed”) model (Paradis 2013) implemented in ape v.4.1. (Paradis et al. 2004) in R 3.3.3 (R Core Team 2017). To calibrate the trees, we used three fossil calibrations that we included as hard lower bounds based on the oldest known fossil of each clade (McMahan et al. 2013): The age of the crown node of Pseudocrenilabrinae was based on the phylogenetic position of the African Eocene fossil †*Mahengechromis* (minimum age: 46 Ma) (Murray 2000, 2001); the MRCA of *Geophagus* + *Gymnogeophagus* was based on †*Gymnogeophagus eocenicus* (minimum age: 40 Ma) (Malabarba et al. 2010); and the MRCA of Cichlasomatini + Heroini was based on the Neotropical †*Plesioheros* and †*Tremembichthys* (minimum age: 40 Ma) (Del Papa 2006; Malabarba and Malabarba 2008; Perez et al. 2010).

### Phylogenetic Discordance among Genomic Markers

We investigated concordance among all gene trees (i.e., gene tree heterogeneity) and between gene trees and the species trees for each genomic marker type. First, we calculated quartet distances (i.e., the number of consistent quartets) between each gene tree in the UCE and exon data sets and their respective concatenated and coalescent-based species trees using the tqDist algorithm and the QuartetStatus function in the R package Quartet v.1.1.0 (Sand et al. 2014; Smith 2019). Second, we calculated gene concordance factors (gCF)—the percentage of gene trees containing a branch of the species tree—and site concordance factors (sCF)—the percentage of decisive sites supporting a particular branch in the species tree—to illustrate disagreement among gene trees and sites and as an alternative measure of nodal support, using the “-gcf” and “-scf” options in IQ-TREE v.2.0.6 (Minh, Hahn, et al. 2020; Minh, Schmidt, et al. 2020). To analyze if the discordance among gene trees or sites is compatible with a neutral ILS model, we carried out χ^2^ tests comparing the number of trees or sites supporting the two discordant topologies—under the assumption of ILS, the discordant topologies should be supported by an equal number of gene trees or sites.

We also explored the strength of support of individual UCE and exon loci for each of the hypothesized species tree topologies following the method of Shen et al. (2017). We calculated and compared site likelihoods for each locus between the ML topology inferred in RAxML as described above and the constrained topologies for each of the species trees inferred using a concatenation or coalescent approach for both genomic data sets at our nodes of interest (supplementary fig. 8, Supplementary Material online). For each site, we calculated the difference in site-wise log-likelihood scores (ΔSLS) as the difference of lnL values between the ML and the alternative species tree hypotheses. ΔSLS is positive when the data support the ML hypothesis and negative when the data fit the alternative topology better. Finally, for each locus, we summed total ΔSLS and obtained a per-locus log-likelihood score (ΔGLS) for each topological comparison.

Discordance between concatenated and coalescent-based species trees can result from anomalous gene trees. Anomalous gene trees may occur under certain conditions of large effective population sizes and short internode branch lengths that create a higher probability for gene trees that do not match the species tree than for gene trees that match (Degnan and Rosenberg 2006). In such cases, concatenation will favor the anomalous gene tree topology, and the branches of the species tree that produce the discrepancies are said to be in the anomaly zone (Kubatko and Degnan 2007). We used the unifying principle of the anomaly zone (Linkem et al. 2016, equation 1) to investigate whether internode branch lengths fall within the theoretical boundaries of the anomaly zone. We used branch lengths in coalescent units from the species trees inferred in ASTRAL for the UCE and exon data sets and implemented the method using the interface available from https://github.com/tkchafin/anomaly_zone.

We tested the hypothesis that phylogenetic discordance is due to introgressive hybridization using Patterson’s *D*-statistic, also known as the ABBA-BABA test (Durand et al. 2011). This test considers three populations or taxa (P1, P2, P3) and an outgroup (O) for which it explores the asymmetry of frequencies of nonconcordant gene trees. In this setting, two allelic patterns: “ABBA” and “BABA,” can appear. In the ABBA pattern, P1 and the outgroup share the ancestral allele “A,” and P2 and P3 share the derived allele, whereas, in the BABA pattern, P2 and the outgroup share the ancestral allele “A” and P1 and P3 have the derived allele “B.” Under the null hypothesis of ILS and absence of gene flow, both patterns should be equally frequent across all gene trees and the *D*-statistic should be zero. Alternatively, an excess of gene trees showing ABBA or BABA patterns is indicative of gene flow between two of the taxa and will respectively result in positive *D* values suggesting introgression between P2 and P3, or negative *D* values suggesting introgression between P1 and P3.

We focused on two sets of relationships that most frequently disagreed between species trees inferred using different genomic markers, and/or inference methods: among the major clades of Heroini and among genera of herichthyine cichlids (i.e., WTC clade, see Results). For testing the relationships among the main clades of Heroini, we included amphilophines, caquetaines, and members of the Greater Antillean endemic genus *Nandopsis*, as our ingroup taxa with the remaining herichthyines as the outgroup. For the WTC clade, the four-taxon test included the genera *Wajpamheros*, *Theraps*, *Chuco*, and *Vieja* as the outgroup. In both cases, we calculated *D*-statistics based on the species trees inferred for each marker type using the R program HybridCheck v.1.0.1 (Ward and van Oosterhout 2016).

## Supplementary Material

Supplementary data are available at *Genome Biology and Evolution* online.

## Supplementary Material

evab161_Supplementary_Data

## Data Availability

Data associated with this manuscript are available under BioProject PRJNA690533, available at http://www.ncbi.nlm.nih.gov/bioproject/690533. Other DNA alignments, analysis inputs, and analysis outputs are available from Alda et al. (2021).
