# Supplementary material for: Comparing Ultraconserved Elements and Exons for Phylogenomic Analyses of Middle American Cichlids: When Data Agree to Disagree
Source: Genome Biol Evol. 2021 Jul 17;13(8):evab161. doi: 10.1093/gbe/evab161 (PMC8369075; doi:10.1093/gbe/evab161)
Supplement: evab161_Supplementary_Data [file evab161_supplementary_data.zip › Aldaetal_GBE_SupplMat.R2.legends.docx]

SUPPLEMENTARY MATERIALS

Supplementary Table 1. List of all samples included in this study and summary statistics of the sequencing output recovered for each one of them.

Supplementary Table 2. List of samples from Ilves et al. (2017) included in the common taxon set.

Supplementary Table 3. Summary of ΔGLS values across UCE and exon loci and for each topological comparison following the method of Shen et al. (2017).

Supplementary Figure 1. Cladogram of the ML tree inferred in RAxML for the complete concatenated dataset of UCEs of all cichlid samples included in this study.

Supplementary Figure 2. Phylogram of the ML tree inferred in RAxML for the complete concatenated dataset of UCEs of all cichlid samples included in this study.

Supplementary Figure 3. Species tree inferred using SVDquartets for the complete dataset of UCEs of all cichlid species included in this study.

Supplementary Figure 4. Topological comparison of the cichlid species trees inferred in RAxML (A) and ASTRAL-III (B) for the complete dataset of UCEs.

Supplementary Figure 5. Topological comparison of the cichlid species trees inferred using SVDquartets for the common taxon set and the UCE (A) and exon (B) datasets complete dataset of UCEs.

Supplementary Figure 6. Scatterplots of gene concordance factor (gCF) values against site concordance factor (sCF) values for all branches in the UCE and exon datasets.

Supplementary Figure 7. Cladograms showing conflicting relationships between the UCE and exon datasets.

Supplementary Figure 8. Cladograms representing the alternative topologies recovered for each marker type and inference method and used in the topology tests following Shen et al. (2017).

Supplementary Figure 9. Plots of per-locus log-likelihood scores (ΔGLS) against the number of parsimony informative sites for each topological test between the ML tree and each of the alternative hypotheses.

Supplementary Figure 10. Cladogram of the ML tree inferred in RAxML for the common taxon set of exon data after removing the 20 exon loci with ΔGLS ≥25.
