# Supplementary material for: Comparing Ultraconserved Elements and Exons for Phylogenomic Analyses of Middle American Cichlids: When Data Agree to Disagree
Source: Genome Biol Evol. 2021 Jul 17;13(8):evab161. doi: 10.1093/gbe/evab161 (PMC8369075; doi:10.1093/gbe/evab161)
Supplement: evab161_Supplementary_Data [file evab161_supplementary_data.zip › Aldaetal_GBE_SupplMat.R2.clean.docx]

Supplementary Materials

Comparing ultraconserved elements and exons for phylogenomic analyses of Middle American cichlids: when data agree to disagree

Fernando Alda^a*^, William B. Ludt^b^, Diego J. Elías^c^, Caleb D. McMahan^d^, Prosanta Chakrabarty^c^

^a^ Department of Biology, Geology and Environmental Science, University of Tennessee at Chattanooga, 615 McCallie Ave., Chattanooga, TN 37403, USA

^b^ Department of Ichthyology, Natural History Museum of Los Angeles County, 900 Exposition Blvd., Los Angeles, CA 90007, USA

^c^ Museum of Natural Science, Department of Biological Sciences, Louisiana State University, 119 Foster Hall, Baton Rouge, LA 70803, USA

^d^ Field Museum of Natural History, 1400 S. Lake Shore Dr., Chicago, IL 60605, USA

*Corresponding author: E-mail: fernando-alda@utc.edu

| **Supplementary Table 1.** List of all samples included in this study and summary statistics of the sequencing output recovered for each one of them. | | | | | | | | | | | | |  |
| --- | --- | --- | --- | --- | --- | --- | --- | --- | --- | --- | --- | --- | --- |
| **Species** | **Tribe: Clade** | **Reads** | **bp** | **Mean contig length** | **sd** | **Min. contig length** | **Max. contig length** | **Median contig length** | **Contigs >1kb** | **Voucher** | **Collection/ Source** | **Common taxon set: Same/Equiv.** | |
| Cichlinae |  |  |  |  |  |  |  |  |  |  |  |  | |
| *Amatitlania altoflava* | Heroini: Amphilophines | 489,817 | 164,290,950 | 335.413 | 0.258 | 78 | 16,609 | 295 | 3,447 | 2147 | LSUMZ |  | |
| *Amatitlania nigrofasciata* | Heroini: Amphilophines | 9,034 | 2,950,876 | 326.641 | 3.272 | 78 | 10,001 | 259 | 239 | 3157 | LSUMZ | E | |
| *Amatitlania septemfasciata* | Heroini: Amphilophines | 554,668 | 177,291,678 | 319.636 | 0.231 | 78 | 16,598 | 289 | 2,817 | 1614 | LSUMZ | E | |
| *Amatitlania siquia* | Heroini: Amphilophines | 7,266 | 2,414,288 | 332.272 | 3.779 | 78 | 10,110 | 259 | 191 | 3004 | LSUMZ | S | |
| *Amphilophus sp.* | Heroini: Amphilophines | 664,010 | 218,192,132 | 328.598 | 0.223 | 78 | 16,599 | 294 | 4,099 | 2921 | LSUMZ |  | |
| *Amphilophus citrinellus* | Heroini: Amphilophines | WGS |  |  |  |  |  |  |  | [PRJEB6974](https://www.ncbi.nlm.nih.gov/bioproject/PRJEB6974) | [GenBank](https://www.ncbi.nlm.nih.gov/nuccore/GCA_000751415.1) | S | |
| *Amphilophus hogaboomorum* | Heroini: Amphilophines | 8,623 | 2,538,566 | 294.395 | 3.044 | 78 | 9,985 | 249 | 157 | 4405 | LSUMZ | S | |
| *Amphilophus istlanus* | Heroini: Amphilophines | 16,344 | 5,086,811 | 311.234 | 1.607 | 78 | 5,338 | 262 | 322 | 2339 | SLU | E | |
| *Amphilophus trimaculatus* | Heroini: Amphilophines | 439,889 | 139,486,642 | 317.095 | 0.240 | 78 | 16,599 | 284 | 2,043 | 2514 | LSUMZ | S | |
| *Archocentrus centrarchus* | Heroini: Amphilophines | 618,577 | 213,799,319 | 345.631 | 0.273 | 78 | 16,714 | 302 | 5,908 | 2850 | LSUMZ | S | |
| *Chortiheros wesseli* | Heroini: Amphilophines | 11,075 | 3,048,752 | 275.282 | 2.966 | 78 | 13,157 | 244 | 195 | 3720 | LSUMZ | S | |
| *Cryptoheros cutteri* | Heroini: Amphilophines | 10,690 | 3,214,785 | 300.728 | 2.896 | 78 | 9,829 | 251 | 209 | 3429 | LSUMZ |  | |
| *Cryptoheros spilurus* | Heroini: Amphilophines | 22,926 | 7,051,215 | 307.564 | 2.030 | 78 | 16,537 | 258 | 468 | 5630 | LSUMZ |  | |
| *Darienheros calobrensis* | Heroini: Amphilophines | 612,372 | 203,555,986 | 332.406 | 0.222 | 77 | 16,554 | 298 | 3,423 | 1930 | LSUMZ | S | |
| *Darienheros calobrensis* | Heroini: Amphilophines | 15,525 | 4,833,069 | 311.309 | 1.637 | 78 | 3,770 | 262 | 291 | 8106 | LSUMZ |  | |
| *Hypsophrys nematopus* | Heroini: Amphilophines | 451,805 | 151,559,662 | 335.454 | 0.285 | 78 | 28,684 | 294 | 3,349 | 1490 | LSUMZ | S | |
| *Hypsophrys nicaraguensis* | Heroini: Amphilophines | 635,625 | 209,507,874 | 329.609 | 0.236 | 78 | 16,600 | 295 | 4,366 | 1499 | LSUMZ | S | |
| *Isthmoheros tuyrensis* | Heroini: Amphilophines | 23,633 | 6,994,223 | 295.952 | 1.238 | 78 | 5,064 | 260 | 359 | 7903 | LSUMZ |  | |
| *Kronoheros umbrifer* | Heroini: Amphilophines | 179,930 | 44,010,631 | 244.599 | 0.384 | 78 | 16,597 | 249 | 836 | 7815 | LSUMZ |  | |
| *Kronoheros umbrifer* | Heroini: Amphilophines | 19,255 | 6,079,241 | 315.723 | 1.542 | 78 | 5,406 | 264 | 545 | 7918 | LSUMZ |  | |
| *Mayaheros beani* | Heroini: Amphilophines | 20,961 | 6,209,694 | 296.250 | 1.109 | 78 | 4,045 | 261 | 142 | KPR8 08A | SLU |  | |
| *Mayaheros urophthalmus* | Heroini: Amphilophines | 27,130 | 7,666,791 | 282.595 | 1.896 | 78 | 16,610 | 253 | 319 | 5830 | LSUMZ | S | |
| *Parachromis dovii* | Heroini: Amphilophines | 370,611 | 111,383,956 | 300.541 | 0.270 | 78 | 16,594 | 275 | 1,910 | 3990 | LSUMZ | S | |
| *Parachromis multifasciatus* | Heroini: Amphilophines | 363,287 | 117,831,811 | 324.349 | 0.312 | 78 | 16,603 | 282 | 3,292 | 5730 | LSUMZ | S | |
| *Parachromis friedrichsthalii* | Heroini: Amphilophines | 300,987 | 89,404,849 | 297.039 | 0.284 | 78 | 16,603 | 273 | 1,273 | 1609 | LSUMZ |  | |
| *Parachromis managuensis* | Heroini: Amphilophines | 538,679 | 186,253,252 | 345.759 | 0.258 | 78 | 16,602 | 302 | 4,834 | 2660 | LSUMZ |  | |
| *Parachromis motaguensis* | Heroini: Amphilophines | 15,490 | 4,718,461 | 304.613 | 2.289 | 78 | 9,722 | 254 | 396 | 2340 | LSUMZ |  | |
| *Petenia splendida* | Heroini: Amphilophines | 370,700 | 112,357,740 | 303.096 | 0.273 | 78 | 16,595 | 276 | 1,835 | 5890 | LSUMZ | S | |
| *Talamancaheros sieboldii* | Heroini: Amphilophines | 435,150 | 142,147,227 | 326.663 | 0.268 | 78 | 16,609 | 289 | 2,858 | 2104 | LSUMZ |  | |
| *Astatheros macracanthus* | Heroini: Astatheroines | 9,034 | 2,613,357 | 289.280 | 2.945 | 78 | 7,486 | 245 | 204 | 2493 | LSUMZ | S | |
| *Cribroheros alfari* | Heroini: Astatheroines | 373,748 | 107,538,522 | 287.730 | 0.271 | 78 | 16,590 | 269 | 1,494 | 4565 | LSUMZ |  | |
| *Cribroheros altifrons* | Heroini: Astatheroines | 813,566 | 233,225,465 | 286.671 | 0.212 | 78 | 16,637 | 269 | 4,451 | 1372 | LSUMZ |  | |
| *Cribroheros bussingi* | Heroini: Astatheroines | 337,193 | 92,233,106 | 273.532 | 0.284 | 78 | 16,615 | 264 | 1,104 | 1700 | LSUMZ |  | |
| *Cribroheros diquis* | Heroini: Astatheroines | 704,774 | 215,724,164 | 306.090 | 0.223 | 78 | 16,633 | 281 | 4,047 | 1343 | LSUMZ |  | |
| *Cribroheros longimanus* | Heroini: Astatheroines | 569,231 | 203,122,472 | 356.837 | 0.255 | 78 | 16,629 | 310 | 5,834 | 4010 | LSUMZ |  | |
| *Cribroheros robertsoni* | Heroini: Astatheroines | 44,446 | 12,517,631 | 281.637 | 1.263 | 78 | 16,636 | 256 | 502 | 5588 | LSUMZ |  | |
| *Cribroheros rostratus* | Heroini: Astatheroines | 192,167 | 54,280,479 | 282.465 | 0.324 | 78 | 16,628 | 265 | 554 | 1588 | LSUMZ |  | |
| *Herotilapia multispinosa* | Heroini: Astatheroines | 562,727 | 172,738,342 | 306.967 | 0.228 | 77 | 16,619 | 281 | 2,589 | 2744 | LSUMZ | S | |
| *Rocio octofasciata* | Heroini: Astatheroines | 17,952 | 5,008,256 | 278.980 | 1.204 | 78 | 3,272 | 257 | 119 | 5 | UMMZ | S | |
| *Tomocichla tuba* | Heroini: Astatheroines | 919,522 | 296,782,892 | 322.758 | 0.215 | 78 | 16,637 | 291 | 8,321 | 1474 | LSUMZ | S | |
| *Caquetaia krausii* | Heroini: Caquetaines | 317,822 | 90,739,166 | 285.503 | 0.254 | 78 | 16,619 | 270 | 890 | 1 | UMMZ | S | |
| *Caquetaia myersi* | Heroini: Caquetaines | 547,515 | 187,322,845 | 342.133 | 0.242 | 78 | 16,739 | 301 | 4,079 | 3730 | LSUMZ |  | |
| *Caquetaia spectabilis* | Heroini: Caquetaines | 209,311 | 60,218,623 | 287.699 | 0.339 | 78 | 16,615 | 267 | 987 | 7813 | LSUMZ | S | |
| *Chiapaheros grammodes* | Heroini: Herychthyines | 24,601 | 6,798,902 | 276.367 | 1.130 | 78 | 7,112 | 257 | 275 | 15 | UMMZ | S | |
| *Chuco godmanni* | Heroini: Herychthyines | 23,381 | 6,012,329 | 257.146 | 1.836 | 78 | 16,616 | 239 | 367 | 5660 | LSUMZ |  | |
| *Chuco intermedius* | Heroini: Herychthyines | 250,892 | 70,489,532 | 280.956 | 0.333 | 78 | 16,617 | 264 | 1,160 | 5740 | LSUMZ | S | |
| *Chuco microphthalmus* | Heroini: Herychthyines | 362,073 | 108,535,496 | 299.761 | 0.275 | 78 | 16,615 | 274 | 1,926 | 3270 | LSUMZ |  | |
| *Cincelichthys bocourti* | Heroini: Herychthyines | 969,689 | 303,532,999 | 313.021 | 0.213 | 78 | 16,628 | 284 | 8,947 | 5575 | LSUMZ |  | |
| *Cincelichthys pearsei* | Heroini: Herychthyines | 393,035 | 116,591,301 | 296.644 | 0.274 | 78 | 17,965 | 273 | 2,080 | 5996 | LSUMZ |  | |
| *Herichthys carpintis* | Heroini: Herychthyines | 12,247 | 3,895,244 | 318.057 | 1.756 | 78 | 3,672 | 264 | 171 | 3271 | SLU | E | |
| *Herichthys cf deppii* | Heroini: Herychthyines | 15,409 | 4,837,337 | 313.929 | 1.609 | 78 | 3,930 | 264 | 286 | 3365 | SLU | E | |
| *Kihnichthys ufermanni* | Heroini: Herychthyines | 26,460 | 7,100,641 | 268.354 | 1.043 | 78 | 6,533 | 255 | 210 | 131789 | FMNH |  | |
| *Maskaheros argenteus* | Heroini: Herychthyines | 32,131 | 9,168,624 | 285.351 | 1.003 | 78 | 7,437 | 260 | 370 | 2310 | UNICACH-MZ |  | |
| *Mesoheros gephyrus* | Heroini: Herychthyines | 460,842 | 126,726,692 | 274.989 | 0.257 | 78 | 16,472 | 264 | 1,691 | 3729 | LSUMZ | S | |
| *Oscura heterospila* | Heroini: Herychthyines | 590,297 | 187,385,721 | 317.443 | 0.233 | 78 | 17,138 | 287 | 3,070 | 6040 | LSUMZ |  | |
| *Paraneetroplus bulleri* | Heroini: Herychthyines | 52,289 | 14,305,540 | 273.586 | 0.663 | 78 | 7,734 | 259 | 382 | 2558 | UNICACH-MZ |  | |
| *Rheoheros lentiginosus* | Heroini: Herychthyines | 664,986 | 205,941,756 | 309.693 | 0.228 | 77 | 16,624 | 282 | 3,884 | 5720 | LSUMZ |  | |
| *Theraps irregularis* | Heroini: Herychthyines | 465,248 | 140,286,223 | 301.530 | 0.255 | 78 | 16,624 | 276 | 2,215 | 6133 | LSUMZ | S | |
| *Thorichthys affinis* | Heroini: Herychthyines | 592,704 | 175,482,685 | 296.071 | 0.229 | 78 | 16,604 | 275 | 2,769 | 5903 | LSUMZ |  | |
| *Thorichthys aureus* | Heroini: Herychthyines | 54,327 | 15,244,108 | 280.599 | 1.106 | 78 | 16,617 | 255 | 619 | 5620 | LSUMZ |  | |
| *Thorichthys helleri* | Heroini: Herychthyines | 496,972 | 144,109,993 | 289.976 | 0.237 | 78 | 16,604 | 271 | 1,825 | 6011 | LSUMZ | S | |
| *Thorichthys meeki* | Heroini: Herychthyines | 438,126 | 124,808,902 | 284.870 | 0.256 | 78 | 16,604 | 268 | 1,823 | 6077 | LSUMZ | S | |
| *Thorichthys pasionis* | Heroini: Herychthyines | 535,304 | 161,369,027 | 301.453 | 0.227 | 78 | 16,610 | 278 | 2,267 | 6083 | LSUMZ |  | |
| *Trichromis salvini* | Heroini: Herychthyines | 31,449 | 9,257,470 | 294.365 | 2.058 | 78 | 23,867 | 254 | 590 | 5653 | LSUMZ | S | |
| *Vieja bifasciata* | Heroini: Herychthyines | 1,298,419 | 4,031,650 | 245.817 | 1.466 | 78 | 3,093 | 244 | 153 | 53 | UMMZ |  | |
| *Vieja fenestrata* | Heroini: Herychthyines | 10,309 | 3,304,372 | 320.533 | 2.088 | 78 | 2,685 | 263 | 192 | 3349 | SLU |  | |
| *Vieja fenestrata* | Heroini: Herychthyines | 18,183 | 5,614,538 | 308.780 | 1.510 | 78 | 6,151 | 262 | 378 | 3361 | SLU |  | |
| *Vieja guttulata* | Heroini: Herychthyines | 340,887 | 95,647,390 | 280.584 | 0.322 | 78 | 27,569 | 266 | 1,569 | 6440 | LSUMZ |  | |
| *Vieja hartwegi* | Heroini: Herychthyines | 49,440 | 13,064,541 | 264.250 | 0.673 | 78 | 7,531 | 256 | 284 | 131457 | FMNH |  | |
| *Vieja maculicauda* | Heroini: Herychthyines | 11,287 | 2,869,574 | 254.237 | 2.778 | 78 | 10,028 | 231 | 198 | 4079 | LSUMZ | S | |
| *Vieja melanura* | Heroini: Herychthyines | 20,975 | 4,920,669 | 234.597 | 1.929 | 78 | 16,642 | 229 | 259 | 5950 | LSUMZ | S | |
| *Wajpamheros nourissati* | Heroini: Herychthyines | 41,760 | 11,961,836 | 286.442 | 0.823 | 78 | 9,384 | 261 | 514 | 131788 | FMNH | S | |
| *Nandopsis haitiensis* | Heroini: *Nandopsis* | 29,425 | 7,802,086 | 265.152 | 0.993 | 78 | 6,051 | 255 | 298 | 56 | UMMZ | S | |
| *Nandopsis haitiensis* | Heroini: *Nandopsis* | 33,303 | 8,854,399 | 265.874 | 0.816 | 78 | 7,588 | 256 | 132 | 6 | UMMZ |  | |
| *Nandopsis tetracanthus* | Heroini: *Nandopsis* | 11,826 | 3,725,310 | 315.010 | 1.980 | 78 | 4,426 | 264 | 263 | 55 | UMMZ | S | |
| *Pterophyllum scalare* | Heroini | 509,883 | 154,191,171 | 302.405 | 0.238 | 78 | 16,570 | 280 | 2,044 | 7351 | LSUMZ | S | |
| *Andinoacara coeruleopunctatus* | Cichlasomatini: Andinoacarines | 172,063 | 50,607,591 | 294.122 | 0.325 | 78 | 16578 | 269 | 348 | 2070 | LSUMZ | E | |
| *Cichlasoma bimaculatum* | Cichlasomatini: Cichlasomatines | 200,377 | 59,824,996 | 298.562 | 0.346 | 78 | 16609 | 269 | 981 | 6518 | LSUMZ | S | |
| *Acarichthys heckelii* | Geophagini: Apistogrammines | UCE |  |  |  |  |  |  |  | [PRJNA396208](https://www.ncbi.nlm.nih.gov/bioproject/PRJNA396208) | Burress et al. (2018) | S | |
| *Apistogramma ortmanni* | Geophagini: Apistogrammines | UCE |  |  |  |  |  |  |  | [PRJNA396208](https://www.ncbi.nlm.nih.gov/bioproject/PRJNA396208) | Burress et al. (2018) | E | |
| *Crenicichla macrophthalma* | Geophagini: Crenicichlines | UCE |  |  |  |  |  |  |  | [PRJNA396208](https://www.ncbi.nlm.nih.gov/bioproject/PRJNA396208) | Burress et al. (2018) | E | |
| *Crenicichla saxatilis* | Geophagini: Crenicichlines | UCE |  |  |  |  |  |  |  | [PRJNA396208](https://www.ncbi.nlm.nih.gov/bioproject/PRJNA396208) | Burress et al. (2018) | E | |
| *Crenicichla sp.* | Geophagini: Crenicichlines | 214,540 | 58,024,377 | 270.459 | 0.332 | 78 | 8,896 | 260 | 781 | 6150 | LSUMZ | E | |
| *Teleocichla gephyrogramma* | Geophagini: Crenicichlines | UCE |  |  |  |  |  |  |  | [PRJNA396208](https://www.ncbi.nlm.nih.gov/bioproject/PRJNA396208) | Burress et al. (2018) | E | |
| *Geophagus crassilabris* | Geophagini: Geophagines | 349,087 | 111,077,003 | 318.193 | 0.304 | 78 | 19,956 | 282 | 1,939 | 1844 | LSUMZ | E | |
| *Gymnogeophagus tiraparae* | Geophagini: Geophagines | UCE |  |  |  |  |  |  |  | [PRJNA396208](https://www.ncbi.nlm.nih.gov/bioproject/PRJNA396208) | Burress et al. (2018) | E | |
| Pseudocrenilabrinae |  |  |  |  |  |  |  |  |  |  |  |  | |
| *Astatotilapia calliptera* | Haplochromini | WGS |  |  |  |  |  |  |  | PRJEB24325 | Malinsky et al. (2015) |  | |
| *Hemichromis letourneuxi* | Hemichromini | 550,512 | 201,804,138 | 366.575 | 0.275 | 78 | 30,791 | 316 | 6,104 | 6520 | LSUMZ | E | |
| *Lamprologus werneri* | Lamprologini | 627,040 | 215,423,453 | 343.556 | 0.262 | 78 | 16,660 | 300 | 6,660 | 6210 | LSUMZ |  | |
| *Neolamprologus brichardi* | Lamprologini | WGS |  |  |  |  |  |  |  | PRJNA60365 | [Brawand et al. (2014)](https://www.ncbi.nlm.nih.gov/pubmed/25186727) |  | |
| *Oreochromis niloticus* | Oreochromini | 308,285 | 88,628,377 | 287.488 | 0.297 | 78 | 16,702 | 267 | 1,486 | 1675 | LSUMZ | S | |
| *Oreochromis niloticus* | Oreochromini | WGS |  |  |  |  |  |  |  | PRJNA59571 | [Brawand et al. (2014)](https://www.ncbi.nlm.nih.gov/pubmed/25186727) |  | |
| Etroplinae |  |  |  |  |  |  |  |  |  |  |  |  | |
| *Etroplus suratensis* |  | 590,180 | 150,757,280 | 255.443 | 0.213 | 78 | 16,501 | 254 | 1,896 | 5136 | LSUMZ | S | |
| Natural history collection codes follow Sabaj (2020). | | | | | | | | | | | | | |
| The common taxon set column indicates whether the species matches the same (S) or an equivalent (E) species in the exon data set. | | | | | | | | | | | | | |
|  | | | | | | | | | | | | | |
| Brawand D et al. 2014. The genomic substrate for adaptive radiation in African cichlid fish. Nature. 513:375–381. | | | | | | | | | | | | | |
| Burress ED et al. 2018. Phylogenomics of pike cichlids (Cichlidae: *Crenicichla*): the rapid ecological speciation of an incipient species flock. J. Evol. Biol. 31:14–30. | | | | | | | | | | | | | |
| Malinsky et al. 2015. Genomic islands of speciation separate cichlid ecomorphs in an East African crater lake. Science. 350:1493-1498 | | | | | | | | | | | | | |
| Sabaj MH. 2020. Codes for natural history collections in ichthyology and herpetology. Copeia. 108: 593-669 | | | | | | | | | | | | | |

| **Supplementary Table 2.** List of samples from Ilves et al. (2017) included in the common taxon set. | | | | |
| --- | --- | --- | --- | --- |
| **Species** | **Tribe: Clade** | **ROM Catalogue No.** | **Common taxon set: Same/Equiv.** |  |
| Cichlinae |  |  |  |  |
| *Amatitlania myrnae* | Heroini: Amphilophines | ROM 83161 | E |  |
| *Amatitlania sajica* | Heroini: Amphilophines | ROM 93804 | E |  |
| *Amatitlania siquia* | Heroini: Amphilophines | ROM 84187 | S |  |
| *Amphilophus citrinellus* | Heroini: Amphilophines | ROM 94566 | S |  |
| *Amphilophus hogaboomorum* | Heroini: Amphilophines | ROM 84157 | S |  |
| *Amphilophus lyonsi* | Heroini: Amphilophines | Photo Voucher | E |  |
| *Amphilophus trimaculatus* | Heroini: Amphilophines | ROM 84164 | S |  |
| *Archocentrus centrarchus* | Heroini: Amphilophines | ROM 83167 | S |  |
| *Chortiheros wesseli* | Heroini: Amphilophines | ROM 84177 | S |  |
| *Darienheros calobrensis* | Heroini: Amphilophines | ROM 84161 | S |  |
| *Hypsophrys nicaraguensis* | Heroini: Amphilophines | ROM 94349 | S |  |
| *Mayaheros urophthalmus* | Heroini: Amphilophines | ECOSUR 5327 | S |  |
| *Neetroplus nematopus* | Heroini: Amphilophines | ROM 83165 | S |  |
| *Parachromis dovii* | Heroini: Amphilophines | ROM 84151 | S |  |
| *Parachromis multifasciatus* | Heroini: Amphilophines | ECOSUR T4306 | S |  |
| *Petenia splendida* | Heroini: Amphilophines | ECOSUR T3706 | S |  |
| *Astatheros macracanthus* | Heroini: Astatheroines | UTFTC 2802 | S |  |
| *Herotilapia multispinosa* | Heroini: Astatheroines | ROM 84137 | S |  |
| *Rocio octofasciata* | Heroini: Astatheroines | ROM 92363 | S |  |
| *Tomocichla tuba* | Heroini: Astatheroines | ROM Uncatalogued | S |  |
| *Caquetaia kraussii* | Heroini: Caquetaines | MCNG Uncatalogued | S |  |
| *Caquetaia spectabilis* | Heroini: Caquetaines | ROM 96102 | S |  |
| *Chiapaheros grammodes* | Heroini: Herychthyines | ROM 84174 | S |  |
| *Chuco intermedius* | Heroini: Herychthyines | ECOSUR T7006 | S |  |
| *Herichthys bartoni* | Heroini: Herychthyines | UTFTC 2600 | E |  |
| *Herichthys cyanoguttatus* | Heroini: Herychthyines | ROM 1822CS | S |  |
| *Mesoheros gephyrus* | Heroini: Herychthyines | ROM 88059 | S |  |
| *Theraps irregularis* | Heroini: Herychthyines | ECOSUR T1509 | S |  |
| *Thorichthys helleri* | Heroini: Herychthyines | ECOSUR T0507 | S |  |
| *Thorichthys meeki* | Heroini: Herychthyines | ECOSUR T0407 | S |  |
| *Trichromis salvini* | Heroini: Herychthyines | ECOSUR Uncatalogued | S |  |
| *Vieja maculicauda* | Heroini: Herychthyines | ROM 84144 | S |  |
| *Vieja melanurus* | Heroini: Herychthyines | Photo Voucher | S |  |
| *Wajpamheros nourissati* | Heroini: Herychthyines | ECOSUR 5338 | S |  |
| *Pterophyllum scalare* | Heroini | ROM 93617 | S |  |
| *Nandopsis haitiensis* | Heroini: *Nandopsis* | ROM 84166 | S |  |
| *Nandopsis tetracanthus* | Heroini: *Nandopsis* | ROM 84162 | S |  |
| *Andinoacara pulcher* | Cichlasomatini: Andinoacarines | ROM 88767 | E |  |
| *Cichlasoma bimaculatum* | Cichlasomatini: Andinoacarines | ROM 83769 | S |  |
| *Acarichthys heckelii* | Geophagini: Apistogrammines | ROM 86511 | S |  |
| *Apistogramma cf steindachneri* | Geophagini: Apistogrammines | ROM 85746 | E |  |
| *Crenicichla geayi* | Geophagini: Crenicichlines | AMNH 235157 | E |  |
| *Crenicichla lugubris* | Geophagini: Crenicichlines | ROM 86128 | E |  |
| *Crenicichla sveni* | Geophagini: Crenicichlines | AMNH 235161 | E |  |
| *Teleocichla preta* | Geophagini: Crenicichlines | ROM 84278 | E |  |
| *Geophagus abalios* | Geophagini: Geophagines | ROM 88270 | E |  |
| *Gymnogeophagus balzanii* | Geophagini: Geophagines | ROM 93805 | E |  |
| Pseudocrenilabrinae |  |  |  |  |
| *Hemichromis cf. bimaculatus* | Hemichromini | AMNH 226519 | E |  |
| *Oreochromis niloticus* | Oreochromini | AMNH254194 | S |  |
| Etroplinae |  |  |  |  |
| *Etroplus suratensis* |  | ROM 93809 | S |  |
| Natural history collection codes follow Sabaj (2020). | | | |  |
| The common taxon set column indicates whether the species matches the same (S) or an equivalent (E) species in the exon data set. | | | |  |

| **Supplementary Table 3.** Summary of ΔGLS values across UCE and exon loci and for each topological comparison following the method of Shen et al. (2017). | | | | | | | |
| --- | --- | --- | --- | --- | --- | --- | --- |
| **UCE** | **Unc_vs_CA2** | **Unc_vs_Heri2** | **Unc_vs_Heri3** | **Unc_vs_Hero2** | **Unc_vs_Hero3** | **Unc_vs_Hero4** | **Unc_vs_Hero5** |
| Mean ΔGLS (sd) | 0.946 (2.432) | -1.192 (3.061) | -1.191 (2.538) | 1.876 (4.172) | 2.404 (4.564) | 1.811 (5.377) | 2.251 (5.354) |
| ΔGLS 95%CI | [0.712, 1.179] | [-1.487, -0.898] | [-1.435, -0.947] | [1.475, 2.277] | [1.965, 2.843] | [1.293, 2.328] | [1.736, 2.766] |
| ΔGLS min - max | -6.991 - 16.341 | -17.161 - 9.377 | -16.661 - 4.817 | -14.051 - 32.986 | -13.726 - 32.233 | -24.881268 - 32.502 | -20.369 - 32.213 |
| No. of loci ΔGLS >1 | 281 (60.300%) | 160 (34.335%) | 156 (33.476%) | 314 (67.382%) | 333 (71.459%) | 298 (63.948%) | 311 (66.738%) |
| No. of loci ΔGLS >15 | 1 (0.215%) | 0 (0.000%) | 0 (0.000%) | 8 (1.717%) | 10 (2.146%) | 15 (3.219%) | 18 (3.863%) |
| No. of loci ΔGLS >25 | 0 (0.000%) | 0 (0.000%) | 0 (0.000%) | 1 (0.215%) | 2 (0.429%) | 3 (0.644%) | 3 (0.644%) |
|  |  |  |  |  |  |  |  |
| **Exon** | **Unc_vs_CA1** | **Unc_vs_Heri1** | **Unc_vs_Heri3** | **Unc_vs_Hero1** | **Unc_vs_Hero2** | **Unc_vs_Hero4** | **Unc_vs_Hero5** |
| Mean ΔGLS (sd) | 0.536 (2.469) | 0.352 (1.767) | 0.000 (0.001) | 4.854 (7.991) | 6.109 (7.737) | 5.497 (9.051) | 6.462 (8.848) |
| ΔGLS 95%CI | [0.299, 0.774] | [0.182, 0.522] | [-0.001, 0.001] | [4.085, 5.623] | [5.365, 6.854] | [4.626, 6.368] | [5.611, 7.313] |
| ΔGLS min - max | -8.891 - 13.838 | -6.017 - 18.172 | -0.004 - 0.008 | -20.903 - 41.696 | -13.914 - 42.628 | -19.771 - 41.516 | -24.881 - 32.502 |
| No. of loci ΔGLS >1 | 248 (59.759%) | 176 (42.410%) | 156 (37.590%) | 304 (73.253%) | 336 (80.964%) | 309 (74.458%) | 324 (78.072%) |
| No. of loci ΔGLS >15 | 0 (0.000%) | 1 (0.241%) | 0 (0.000%) | 39 (9.398%) | 48 (11.566%) | 56 (13.494%) | 64 (15.422%) |
| No. of loci ΔGLS >25 | 0 (0.000%) | 0 (0.000%) | 0 (0.000%) | 9 (2.169%) | 11 (2.651%) | 17 (4.096%) | 19 (4.578%) |

**Supplementary Fig. 1.** Cladogram of the ML tree inferred in RAxML for the complete concatenated data set of UCEs of all cichlid samples included in this study. All nodes are supported by bootstrap values = 100, unless noted. Fish illustration: *Chuco intermedium*.

**Supplementary Fig. 2.** Phylogram of the ML tree inferred in RAxML for the complete concatenated data set of UCEs of all cichlid samples included in this study. All nodes are supported by bootstrap values = 100, unless noted. Fish illustration: *Thorichthys meeki*.

**Supplementary Fig. 3.** Species tree inferred using SVDquartets for the complete data set of UCEs of all cichlid species included in this study. All nodes are supported by bootstrap values = 100. Fish illustration: *Theraps nourissati*.

**Supplementary Fig. 4.** Topological comparison of the cichlid species trees inferred in RAxML (A) and ASTRAL-III (B) for the complete data set of UCEs.


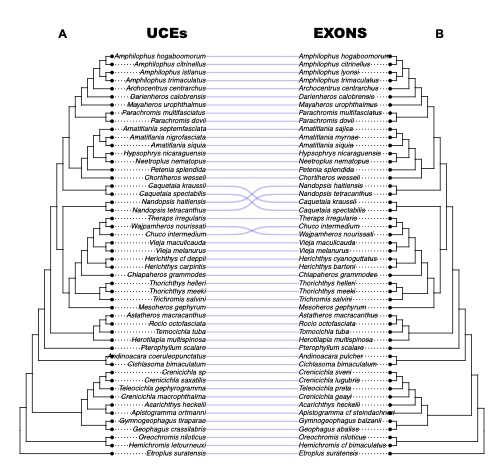


**Supplementary Fig. 5.** Topological comparison of the cichlid species trees inferred using SVDquartets for the common taxon set and the UCE (A) and exon (B) data sets complete data set of UCEs. All nodes are supported by bootstrap values = 100.

**Supplementary Fig. 6.** Scatterplots of gene concordance factor (gCF) values against site concordance factor (sCF) values for all branches in the UCE and exon data sets. Data points are color coded based on the bootstrap value of the corresponding branch.

**Supplementary Fig. 7.** Cladograms showing conflicting relationships between the UCE and exon data sets. The tables below indicate the gene concordance factor (gCF) and site concordance factor (sCF) values for the most frequent quartet (in the figure) and the two alternative quartets, and the absolute number of concordant gene trees (gN) and sites (sN) for the most frequent and alternative topologies calculated based on the number of decisive gene trees and sites for each branch (Minh et al. 2020). Asterisks indicate significant (* *P* < 0.05, ** *P* < 0.01) differences in the frequencies of genes or sites supporting the alternative tree topologies that reject the null hypothesis of ILS as the only source of gene tree disagreement.

**
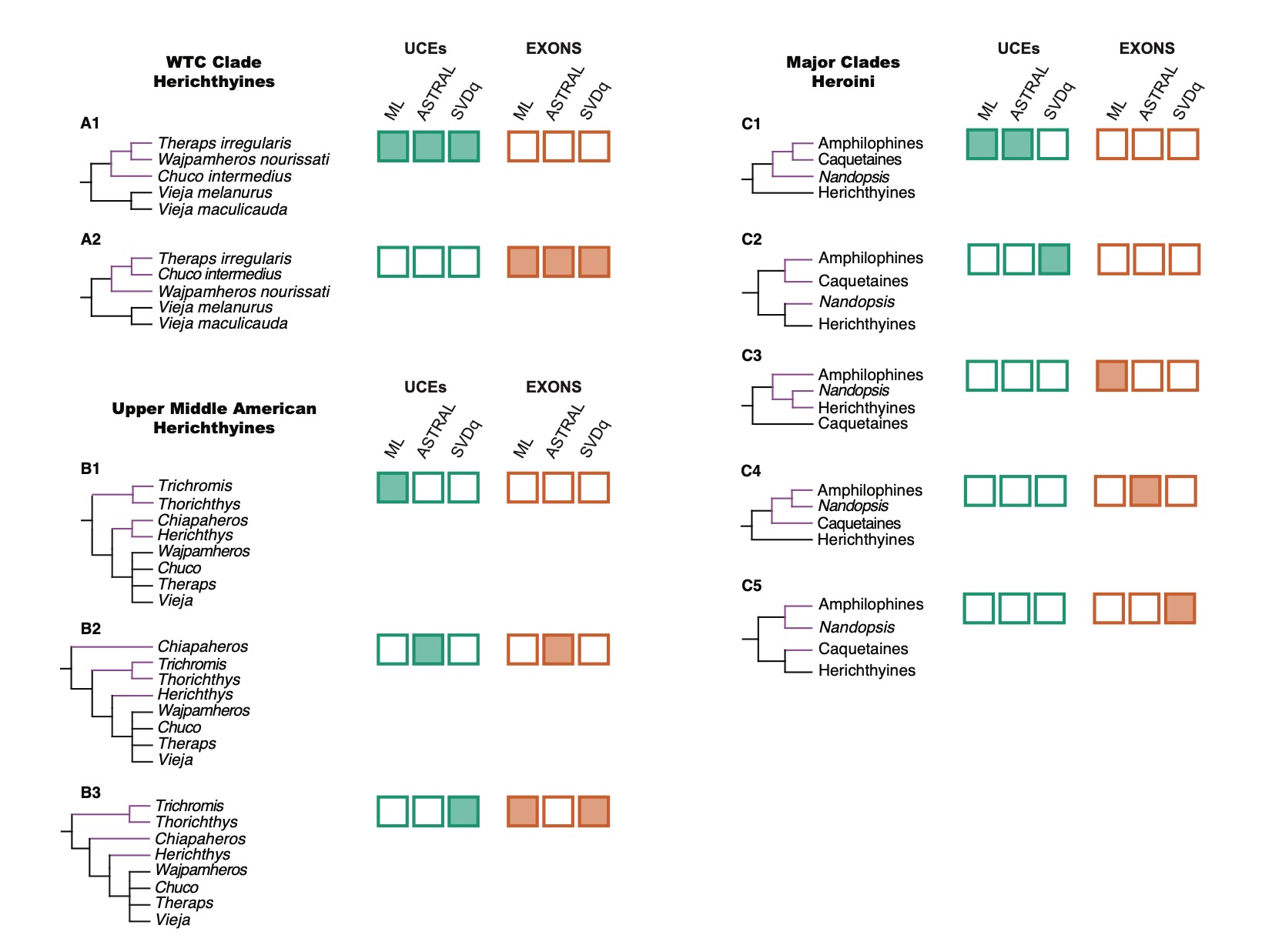
**

**Supplementary Fig. 8.** Cladograms representing the alternative topologies recovered for each marker type and inference method and used in the topology tests following Shen et al. (2017).


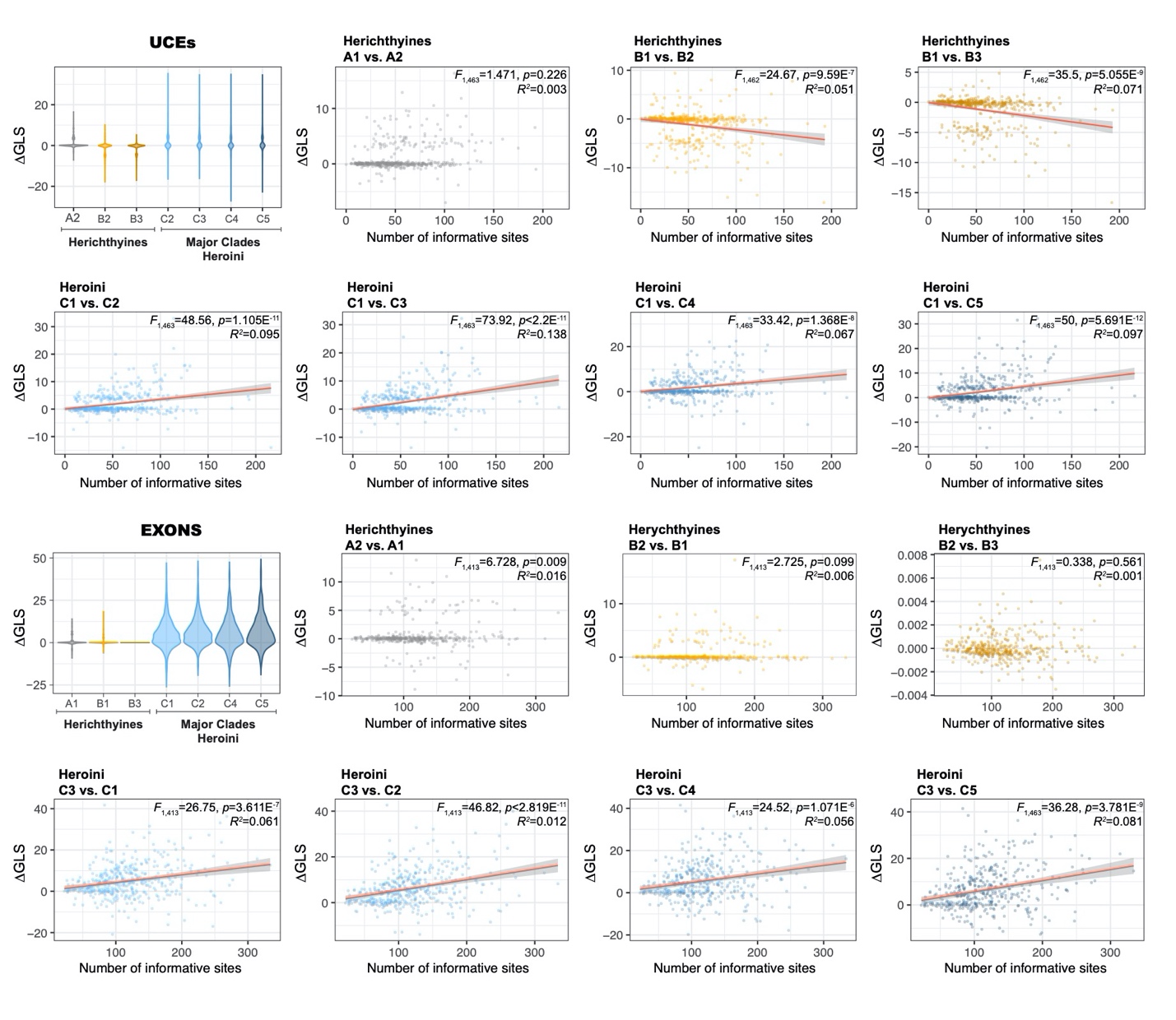


**Supplementary Fig. 9.** Plots of per-locus log-likelihood scores (ΔGLS) against the number of parsimony informative sites for each topological test between the ML tree and each of the alternative hypotheses. See Supplementary Fig. 8.

**Supplementary Fig. 10.** Cladogram of the ML tree inferred in RAxML for the common taxon set of exon data after removing the 20 exon loci with ΔGLS ≥25. All nodes are supported by bootstrap values = 100, unless noted. Fish illustration: *Parachromis friedrichsthalii*.
